# Programmable photonic neural networks combining WDM with coherent linear optics

**DOI:** 10.1038/s41598-022-09370-y

**Published:** 2022-04-04

**Authors:** Angelina Totovic, George Giamougiannis, Apostolos Tsakyridis, David Lazovsky, Nikos Pleros

**Affiliations:** 1 Department of Informatics, Center for Interdisciplinary Research and Innovation - CIRI, Aristotle University of Thessaloniki, Balkan Center - Building A, 10th Km Thessalonikis-Thermis Av, 57001 Thessaloníki, Greece; 2 Celestial AI, 3001 Tasman Drive, Santa Clara, CA 95054 USA

**Keywords:** Electrical and electronic engineering, Integrated optics, Optoelectronic devices and components, Information theory and computation

## Abstract

Neuromorphic photonics has relied so far either solely on coherent or Wavelength-Division-Multiplexing (WDM) designs for enabling dot-product or vector-by-matrix multiplication, which has led to an impressive variety of architectures. Here, we go a step further and employ WDM for enriching the layout with parallelization capabilities across fan-in and/or weighting stages instead of serving the computational purpose and present, for the first time, a neuron architecture that combines coherent optics with WDM towards a multifunctional programmable neural network platform. Our reconfigurable platform accommodates four different operational modes over the same photonic hardware, supporting multi-layer, convolutional, fully-connected and power-saving layers. We validate mathematically the successful performance along all four operational modes, taking into account crosstalk, channel spacing and spectral dependence of the critical optical elements, concluding to a reliable operation with MAC relative error $< 2\%$.

## Introduction

The explosive growth of Artificial Intelligence (AI) and Deep Learning (DL) together with maturing photonic integration have created a new window of opportunity for use of optics in computational tasks^1–5^. The use of photons and relevant optical technologies in Neural Network (NN) hardware is predicted to offer a significant boost in Multiply-Accumulate (MAC) operations per second compared to the respective NN electronic platforms, with computational energy and area efficiency being estimated to reach < fJ/MAC and > TMAC/s/mm$^{2}$, respectively^6,7^. The pathway towards realizing this NN hardware paradigm-shift aims to exploit the high line-rates supported by integrated photonic technologies together with the small-size and low-power weighting function that can be offered at chip-scale^4,8^. So far, the vast majority of photonic devices utilized for weighting purposes has emphasized on slowly reconfigurable elements, like Thermo-Optic (T/O) phase shifters^9,10^ and Phase-Change Material (PCM)-based non-volatile memory structures^4,8^, implying that inference applications are currently considered as the main target within the area of neuromorphic photonics^3^.

Inference engines indeed require a rather static neuron architecture and a layer connectivity graph that usually gets defined for optimally performing a certain AI task. Object tracking and image classification, for example, are typically performed via a number of convolutional layers followed by one or more Fully Connected (FC) layers, while autoencoders require cascaded stages of FC layers^11,12^. Although convolutional and FC layers comprise critical architectural elements in almost all inference platforms, a large set of parameters—such as the number of layers and/or neurons per layer and the connectivity graph—can vary significantly depending on the targeted DL architecture and application. Electronic implementations may conclude to Application-Specific Integrated Circuits (ASICs) customized for a specific inference task, but the use of GPUs, TPUs or even FPGAs becomes unavoidable when reprogrammability and reconfigurability are required in order to utilize the same hardware for multiple applications^13^.

Transferring the reconfiguration capability to Photonic (P)-NN implementations requires a platform that can flexibly support different functional layouts over the same neural hardware. Programmability in photonics has made significant progress over the last years^14–16^ and programmable Photonic Integrated Circuits (PICs) have been shown to offer important advantages towards releasing cost-efficient, flexible and multi-functional photonic platforms that can closely follow the concept of electronic FPGAs^17^. In this effort, it has also been highlighted that just the use of slowly reconfigurable $2 \times 2$ Mach-Zehnder Interferometric (MZI) switches within an appropriate architectural scheme can yield a large set of circuit connectivities and functionality options^14,15^. However, the idiosyncrasy of NN architectures has to proceed along alternative functionalities that are currently still not offered by programmable photonic implementations. Although weight value reconfiguration can be indeed offered by state-of-the-art photonic weighting technology^4,8–10^ and a shift in perspective towards programmable activation functions has also started to emerge^16,18,19^, neuromorphic photonic architectures demonstrated so far are not supporting any reconfiguration mechanism for their linear neuron stages. PNNs have so far progressed along two main architectural categories for realizing linear neural layers, where Wavelength-Division-Multiplexed (WDM) and coherent platforms seem to follow discrete and parallel roadmaps: (i) incoherent or WDM-based layouts, where a discrete wavelength is used for each axon within the same neuron^3,4,20^, and (ii) coherent interferometric schemes, where a single wavelength is utilized across the entire neuron, exploiting interference between coherent electrical fields for weighted sum operations^9,10^.

Here, we present a novel architecture that can efficiently combine WDM and coherent photonics towards supporting Programmable PNNs (PPNNs) with four different linear neural layer operational modes. Starting from our recently proposed dual-IQ coherent linear neuron architecture^21^, that has been recently demonstrated also as a PIC with the ground breaking compute-rates per axon^22,23^, we extend single neuron architecture by employing multiple wavelength channels and respective WDM De/Multiplexing (DE/MUX) structures towards creating multi- and single-element fan-in (input) and weight stages per every axon. Programmability is then enforced through $2 \times 2$ MZI switches that can flexibly define the connectivity between fan-in and weighting stages, allowing in this way for software-defined neural layer topologies. We formulate the mathematical framework for this programmable neuromorphic architecture and proceed with an in-depth study of the anticipated performance impairments originating from the use of multiple wavelengths within the same interferometric arrangement. We conclude to a simple mechanism for counteracting wavelength-dependent behaviour of modulators and phase shifters at the fan-in and weighting stage, respectively, showing that our programmable layout performs equally well for any number of employed optical channels in any of the 4 distinct modes of operation, with all supported neurons always offering a relative error lower than $2\%$ as long as the inter-channel crosstalk is kept at typical values of less than $-\,20 \, \mathrm {dB}$.

## PPNN architecture and operating principle

**Figure 1:**
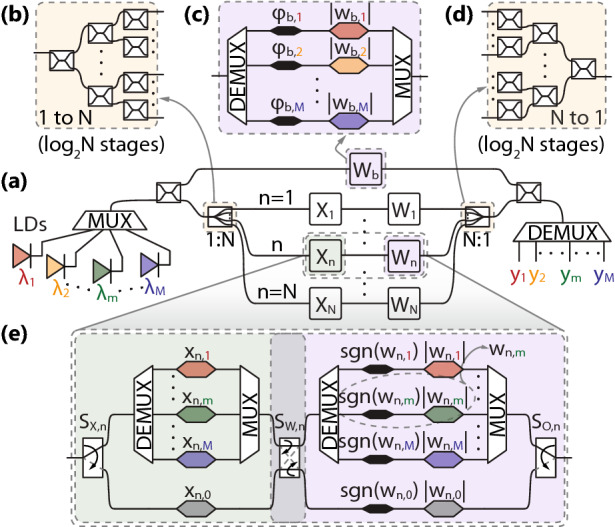
(**a**) Schematic representation of PPNN showing *M* laser diodes (LDs), a MUX, a 3dB X-splitter followed by a bias branch ($W_\mathrm {b}$) and a reconfigurable OLAU encompassing 1-to-*N* splitting stage, input ($X_n$) and weight ($W_n$) modulator banks and an *N*-to-1 combiner stage, the output of which is brought to interfere with the bias signal within 3dB X-coupler and sent to the DEMUX. Closer look into (**b**) 1-to-*N* splitting and (**d**) its $\pi$-rotated *N*-to-1 coupling stage. Zoom-in into the (**c**) bias branch wavelength selective weights and phase modulators and (**e**) an axon of the OLAU consisting of switches for signal routing and modulators for inputs ($x_{n,m}$) and weights ($w_{n,m}$).

In our recent study^21^ we have demonstrated how coherent linear neurons, offering dot-product functionality, can be constructed of IQ-modulator blocks, allowing for the sign information (encoded into the signal’s phase) to be preserved by introducing the biasing signal, $\Sigma w_i x_i + b$, making the neuron compatible with all-optical nonlinear activation functions, $f_\mathrm {NL}(\cdot )$, tailored either for electric field, or for optical power, without suffering information loss. Having the wavelength domain unexploited, we advance our original neuron architecture in order to accommodate multiple channels and achieve parallelization as shown in Fig. 1.

As Fig. 1a reveals, the backbone of our neural layer remains similar as in^21^ with the main differences being: (i) a single Continuous Wave (CW) input optical signal is now replaced by *M* multiplexed CW signals, each centered at $\lambda _m$ and supporting one independent virtual neuron, and (ii) input and weight modulators are now replaced by more elaborate modulator banks given in Fig. 1c, e, delimited by software-controllable switches in the case of latter. The input, multichannel signal is first split by a 3dB X-coupler to the portion directed to the bias branch and the remaining one entering the Optical Linear Algebraic Unit (OLAU). Within the OLAU, the signal gets further split equally in terms of power by a 1-to-*N* splitter, an example of which is given in Fig. 1b, and, after being appropriately modulated by inputs $x_{n,m}$ and pondered by weights $w_{n,m}$, gets sent to the *N*-to-1 combiner, shown in Fig. 1d. At this stage, the output signal interferes with the bias within a 3dB X-coupler and is forwarded to the DEMUX to generate the outputs $y_m$. Finally, each channel *m* will have its own algebraic addition of the weighted inputs with a designated bias, concluding to a total of *M* independent *N*-fan-in neurons.

**Table 1:** PPNN modes of operation and the corresponding switch states.

	Mode	$S_{\mathrm {X},n}$	$S_{\mathrm {W},n}$	$S_{\mathrm {O},n}$
#1	Multi-neuron	1 (up)	1 (bar)	1 (up)
#2	Convolutional	1 (up)	0 (cross)	0 (down)
#3	Fully-connected	0 (down)	0 (cross)	1 (up)
#4	Power-saving	0 (down)	1 (bar)	0 (down)

**Figure 2:**
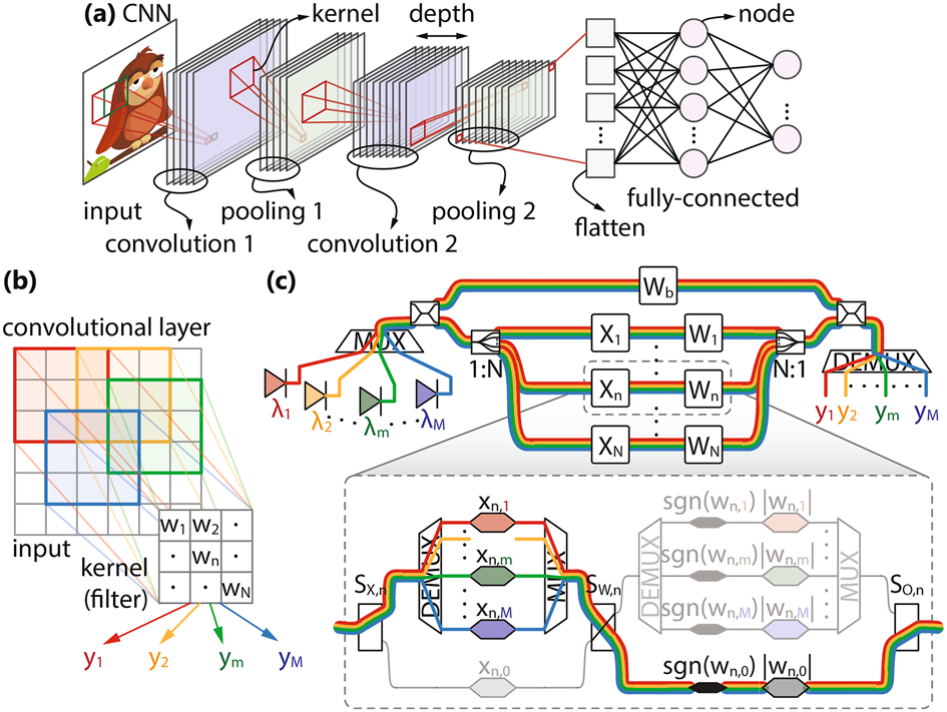
(**a**) Simplified CNN inspired by LeNet-5, employed in image classification. (**b**) Schematic of a convolutional layer with color coded input/output pairs and (**c**) its implementation over PPNN in mode #2 where each channel *m* corresponds to one input/output pair.

**Figure 3:**
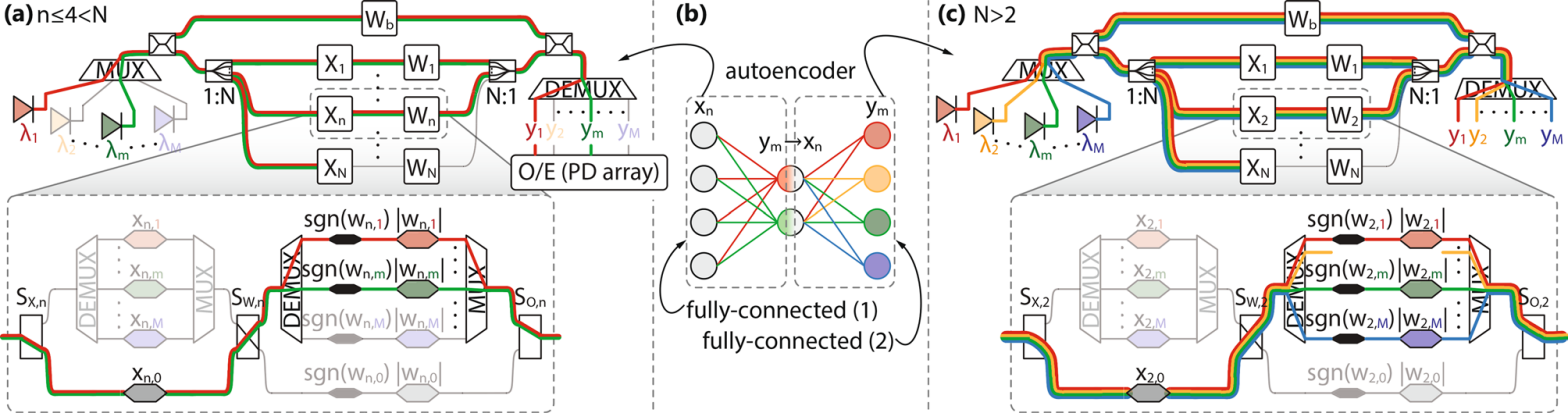
(**b**) Schematic of an autoencoder and (**a**), (**c**) its two FC layers implemented over PPNN in mode #3 where channels correspond to unique weight vectors and outputs $y_m$. Based on the connectivity graph from (**b**), the implementation assumes the use of (**a**) 4 branches and 2 wavelengths in the first layer and (**c**) 2 branches and 4 wavelengths in the second one. If the number of available branches *N* is greater than needed, all the excess branches will have the inputs set to 0 (observe the *N*th branch in (**a**), (**c**), where the condition $N>4$ and $N>2$ is imposed, respectively). Index *n* in the implementation (**a**) is set to $n \le 4$ to denote that the lit *n*th branch carries a non-zero input. Similarly, if the number of available wavelengths *M* exceeds the number of required ones, the excess LDs are powered off.

Depending on the configuration of switches, an overview of which is given in Table 1, channels within a single axon from Fig. 1e, can be controlled either individually or by a common modulator, allowing the network to operate as:

1. *multi-neuron* (*M* independent *N*-to-1 neurons), allowing for an arbitrary logical interconnection graph, supporting even a multi-layer operation by designating different neurons to different layers of the NN;
2. *convolutional* (*M* independent *N*-element inputs with a single kernel of size *N*), where all different input vectors pass through the same set of weights, Fig. 2c, achieving simultaneous *M*-fold usage of the same kernel, speeding up convolution operation from Fig. 2b;
3. *fully-connected* (FC) (single *N*-element input over *M* neurons), where a single input passes through all *M* available weight sets, each of size *N*, allowing for full connectivity between all inputs and outputs, Fig. 3a, c;
4. *power-saving* (single *N*-to-1 neuron), which, even though is not a primarily targeted mode of operation due to large footprint penalty and low aggregated throughput, still allows for resource conservation by powering-off the excess channels and can be useful if NN is occasionally required to operate in sequential manner (one neuron at a time).

A detailed mapping between the architecture from Fig. 1 and the enlisted modes of operation can be found in Section 1, Supplementary Document, with some examples also given in Figs. 2 and 3. Convolutional and FC modes of operation are particularly important due to their ubiquitous presence in deep NNs, especially in the widely-used Convolutional NNs (CNNs), Fig. 2a^11^. In both convolutional and pooling layers, a unique kernel (filtering or weighting window) is applied to the inputs in a scanning manner with a certain stride, yielding a single output value, as depicted schematically in Fig. 2b and implemented over PPNN in Fig. 2c. On the other hand, FC layer, shown implemented over PPNN in Fig. 3a, c, has a single set of inputs passing through multiple sets of weights to produce the outputs and it is the main building block of autoencoders, Fig. 3b, along with being necessary in CNNs, Fig. 2a. Both of these operations are time and energy consuming if approached to in a sequential manner, implying that they greatly benefit from parallelization.

**Table 2:** Input and weight matrices of the *n*th axon.

Mode	$X_n$	$W_n$
#1	diag$[x_{n,1}, \ldots , x_{n,M}]$	diag$[w_{n,1}, \ldots , w_{n,M}]$
#2	diag$[x_{n,1}, \ldots , x_{n,M}]$	$w_{n,0} I_M$
#3	$x_{n,0} I_M$	diag$[w_{n,1}, \ldots , w_{n,M}]$
#4	$x_{n,0} I_M$	$w_{n,0} I_M$

Although the switches of different axons can be controlled independently, the resulting mixed type NN layer has no application foreseen at the moment. Therefore, we assume that switches in all branches are synchronized in the following manner $S_{\mathrm {X},n} = S_\mathrm {X}$, $S_{\mathrm {W},n} = S_\mathrm {W}$ and $S_{\mathrm {O},n} = S_\mathrm {O}, \forall n$. The matrices encapsulating the values of the inputs, $X_n$, and weights, $W_n$, for different modes of operation are summarized in Table 2 where $I_M$ stands for $M \times M$ identity matrix. Inputs require no more than one amplitude modulator per value, since they are defined on the positive domain $x_{n,m} \in [0,1]$, whereas, in case of weights, which can be both positive and negative, $w_{n,m} \in [-1,1]$, two modulators are required, one for the amplitude, which will be proportional to the weight magnitude, $|w_{n,m}|$, and the remaining for the phase, which will be carrying the sign of the weight, $\varphi _{n,m} = [1 - \mathrm {sgn}(w_{n,m})]\pi /2$.

The bias branch, given in Fig. 1c differs from the axon branch, Fig. 1e, in two aspects: (i) it has no input sequence modulator(s); (ii) it has only one possible route the signal can take, with a separate control of each channels’ phase and amplitude. The latter comes as a counteraction measure to the anticipated wavelength-dependent variation of the input and weight magnitudes when a single phase- and amplitude-modulator is used in each axon of the OLAU. Moreover, it allows for compensating potentially different transmission coefficients and phase offsets that will be accumulated by different channels within OLAU, therefore meeting the conditions for constructive interference at the last 3dB coupler of the PNN. Bias matrix remains the same for all modes of operation and reads $W_\mathrm {b} = \mathrm {diag} [w_{\mathrm {b},1}, \ldots , w_{\mathrm {b},M}]$, where $w_{\mathrm {b},m} = |w_{\mathrm {b},m}| \exp ( i\varphi _{\mathrm {b},m} )$.

Let us assume that the optical carrier consists of *M* channels $\lambda _m$, and is represented via an $M \times 1$ column-vector of electric fields $\mathrm {E}_\mathrm {LD} = [E_{\mathrm {LD},1}, \ldots , E_{\mathrm {LD},M}]^\mathrm {T}$, which are normalized such that their magnitude squared yields optical power, i.e., $E_{\mathrm {LD},m} = \sqrt{P_{\mathrm {LD},m}} \exp (i \varphi _{\mathrm {LD},m})$. Following the architecture given in Fig. 1 and the detailed derivation presented in Section 2 of Supplementary Document, we find the column-vector of electric fields at the output of PPNN as

1 $$\begin{aligned} \mathrm {E}_\mathrm {out} = \frac{1}{2} \left( \mathrm {e}^{ i \pi /2 } \right) ^{1 + \log _2 N } \left( {\widetilde{W}}_\mathrm {b} + \frac{1}{N} \sum _{n = 1}^{N} W_n X_n \right) \times \mathrm {E}_\mathrm {LD} \, , \end{aligned}$$

where, in order to ensure constructive interference at the last 3dB X-coupler of Fig. 1a, phase matching between the bias and the signal coming from OLAU is performed. The former is done through ${\widetilde{W}}_\mathrm {b} = W_\mathrm {b} \exp (-i \pi /2)^{\log _2 N}$, which denotes the bias branch channel-wise transfer matrix accounting for phase alignment, with its *m*th element being ${\widetilde{w}}_{\mathrm {b},m} = |w_{\mathrm {b},m}| \exp ( i\varphi _{\mathrm {b},m} ) \exp (-i \pi /2)^{\log _2 N}$. Disregarding accumulated phase shift and losses that are identical for all channels, the transfer matrix of the PPNN, $\mathrm {Q}_\mathrm {t}$, can be written as

2a $$\begin{aligned} \mathrm {Q}_\mathrm {t}&= \mathrm {diag} [ q_{\mathrm {t},1}, \ldots , q_{\mathrm {t},M} ] = {\widetilde{W}}_\mathrm {b} + \frac{1}{N} \sum _{n = 1}^{N} W_n X_n \, , \end{aligned}$$

2b $$\begin{aligned} q_{\mathrm {t},m}&= {\widetilde{w}}_{\mathrm {b},m} + \frac{1}{N} \sum _{n = 1}^{N} w_{n,m} x_{n,m} \, . \end{aligned}$$

The *m*th element of $\mathrm {Q}_\mathrm {t}$ matrix, $q_{\mathrm {t},m}$, given by Eq. (2b) for multi-neuron mode of operation (#1), reveals the underlying principle of operation of our PPNN, demonstrating how normalized dot-product between the *N*-element vectors represented across axons, $[w_{1,m}, \ldots , w_{N,m}]$ and $[x_{1,m}, \ldots , x_{N,m}]$, can be achieved at the *m*th channel neuron output with bias ${\widetilde{w}}_{\mathrm {b},m}$ superimposed to it. The reconfigurability of PPNN is concealed in Eq. (2a), where the choice of matrices $X_n$ and $W_n$ is governed by the mode of operation according to the Table 2, leading to alternative functionalities. In convolutional mode (#2), a single kernel as in Fig. 2b, i.e., a single set of weights across different channels $[w_{1,0}, \ldots , w_{N,0}]$, calls for common weight modulator per axon since $w_{n,m} = w_{n,0}, \forall m$, whereas the input vectors remain different across the channels, $[x_{1,m}, \ldots , x_{N,m}]$, concluding to *M*-fold parallelization, and consequently acceleration, of convolution operation. On the other hand, in FC mode (#3), a single input vector $[x_{1,0}, \ldots , x_{N,0}]$, calling for one input modulator $x_{n,0}$ per *n*th axon, is passed through multiple, channel selective, weights, $[w_{1,m}, \ldots , w_{N,m}]$, yielding full connectivity between all *N* inputs $x_{n,0}$ and all *M* outputs $y_m$, as depicted in Fig. 3b. Finally, in power-saving mode (#4), unique weight and input vectors, $[w_{1,0}, \ldots , w_{N,0}]$ and $[x_{1,0}, \ldots , x_{N,0}]$, allow for only one channel to be used and the remaining ones to be powered off, offering the same functionality as our dual-IQ dot-product engine from^21^ without additional penalties in power consumption or throughput per channel, albeit, suffering from footprint penalty imposed by PPNN programmability and multi-channel design. This mode of operation is certainly not the preferred one, but, in case reconfigurability is a necessary feature of the system, such as in prototyping stages, one can save power when faced with sequential operations, typically embracing the parallel ones, in the form of setup and analysis procedures.

As noted earlier, Eq. (2b) is given for mode #1, but can be updated to any other by replacing the channel-specific $x_{n,m}$ and/or $w_{n,m}$, by a joint $x_{n,0}$ and/or $w_{n,0}$. In what follows, except when explicitly noted otherwise, we will be using $x_{n,m}$ and $w_{n,m}$ notation for an arbitrary mode of operation for simplicity and clarity.

In certain application scenarios, such as image classification, Fig. 2a, b, it is convenient to choose the number of axons as a square of the linear filter (kernel) dimension which is typically an odd number, resulting in, e.g., $N = 3 \times 3$ or $N = 5 \times 5$. Some other applications may call for an arbitrary *N*, not necessarily a square. In this case two approaches can be adopted to exploit the PPNN architecture from Fig. 1, bearing in mind that splitter and combiner from Fig. 1b, d were engineered assuming *N* to be a power of 2. First approach is straight-forward and assumes using the *N* needed axons and ignoring the remaining ones that are supplementing to the closest power-of-2 number larger than *N*. In this case, certain amount of optical power will be lost, but being proportional to $N/ 2^{ \lceil \log _2 N \rceil }$, loss will never exceed 3dB. Second approach aims to eliminate power losses at the expense of redesigning the splitter and combiner, asserting identical phase shift along all paths resulting in coherence preservation between the signals traveling along different axons. The algorithm for designing such splitter and the corresponding combiner is presented in Section 3 of Supplementary Document.

## Impairment analysis

Operating PPNN in power-saving mode with a single active channel, opens the possibility to bypass the DE/MUXes in axons and center all passive (splitters, combiners) and active components (switches, input and weight modulators) to the channel’s central wavelength, leaving no room for output degradation due to wavelength dependent properties of optical components. On the other hand, having a multichannel PPNN (modes #1 through #3) rightfully raises a concern on whether all channels will perform in equal manner, having similar relative error between the targeted output, given by matrix element $q_{\mathrm {t},m}$ in Eq. (2b), and experimentally obtained value $q_{\mathrm {e},m}$. The wavelength dependent loss and phase accumulation along with the crosstalk in DE/MUXes could lead to performance degradation of some channels to a higher extent than the others, measured by increase of absolute, $\Delta q_m = q_{\mathrm {e},m} - q_{\mathrm {t},m}$, and relative error, $\delta q_m = |\Delta q_m|/q_{\mathrm {t},m}$, between the matrix elements. Setting the limit for tolerable relative error can be a challenging task as the network’s error-tolerance depends on the assignment in which it is employed and on the training algorithm. As a rule of thumb, an acceptable PPNN error should be lower than the training error, which is commonly in the range of few percent^21–23^. Moreover, employing noise-aware training algorithms has proven to increase the resilience of the NN models even in the noisy environment^24^, where the noise should be understood as a broad term encapsulating any randomly distributed deviation from the targeted output. Following the above said, in this Section we set to investigate how much will the experimental PPNN transfer matrix, $\mathrm {Q}_\mathrm {e}$, deviate from the targeted one, $\mathrm {Q}_\mathrm {t}$, and whether this deviation can be counteracted.

We start our analysis by examining the effect of wavelength dependence of X-couplers, used for splitting and combining stages, as well as optical switches, used for signal routing within the axons. In what follows, the number of axons *N* is assumed to be a power of two, implying that the splitting and combining stages are composed of cascaded 3dB X-couplers. Nevertheless, all the conclusions can be generalized to an arbitrary number of axons *N*, following the splitter/combiner design outlined in Section 3 of Supplementary Document. The wavelength dependent power splitting ratio of the coupler for the *m*th channel can be written as $\alpha _m = 1/2 + \Delta \alpha _m$, where $\Delta \alpha _m$ denotes coefficient’s deviation from the targeted value of 1/2. All three switches, $S_\mathrm {X}$, $S_\mathrm {W}$ and $S_\mathrm {O}$, are assumed to introduce wavelength dependent loss-penalty, such that the amount of optical power forwarded to the active port is proportional to $s_m \le 1$. According to the detailed study reported in Section 4 of Supplementary Document, we find the output electric field from PPNN in a column-vector form

3 $$\begin{aligned} \mathrm {E}_\mathrm {out} = \frac{1}{2} \mathrm {S}^3 \left( \mathrm {A}_\mathrm {bar} \mathrm {A}_\mathrm {cross} \mathrm {e}^{ i \pi /2 } \right) ^{1 + \log _2 N} \left( {\widetilde{W}}_\mathrm {b} + \frac{1}{N} \sum _{n = 1}^{N} W_n X_n \right) \times \mathrm {E}_\mathrm {LD} \, , \end{aligned}$$

where $\mathrm {S} = \mathrm {diag} \left[ \sqrt{s_1} , \ldots , \sqrt{s_M} \right]$ denotes the transfer matrix of the switch and $\mathrm {A}_\mathrm {bar/cross} = \mathrm {diag} \left[ \sqrt{1 \mp 2 \Delta \alpha _1} , \ldots , \sqrt{1 \mp 2 \Delta \alpha _M} \right]$ stands for the bar/cross transfer matrix of an X-coupler, both wavelength dependent. Ensuring the constructive interference at the output 3dB coupler and preserving the sign integrity of the resulting output field requires phase compensation and per-channel loss balancing within the bias branch, which is achieved by modified weight matrix ${\widetilde{W}}_\mathrm {b}$, with its *m*th element

4 $$\begin{aligned} {\widetilde{w}}_{\mathrm {b},m} = s_m^{-3/2} \left( \sqrt{ 1 - 4 \Delta \alpha _m^2 } \mathrm {e}^{ i \pi /2 } \right) ^{- \log _2 N} w_{\mathrm {b},m} \, . \end{aligned}$$

Both the coefficient pondering $w_{\mathrm {b},m}$ in (4) and the one pondering $\mathrm {Q}_\mathrm {t}$ in (3) depend only on the properties of the switches and X-couplers, and remain unchanged regardless of the input sequence and/or weighs. Comparing (3) to the ideal case given by (1)–(2), it can be seen that the interference condition is successfully fulfilled by individual control of the bias amplitude and phase according to (4). Different channels will certainly accumulate different amount of loss, however, this disbalance can be easily counteracted by employing a set of Variable Optical Attenuators (VOAs) at the demultiplexed output of the PPNN (refer to Fig. 1a). Having the possibility to resolve this challenge outside of the core of PPNN, from this point on, we assume that wavelength dependence of X-couplers and switches is not critical, and we focus on the impairments which may cause degradation of the targeted matrix $\mathrm {Q}_\mathrm {t}$.

For implementing the inputs $x_{n,c}$, we use Mach-Zehnder Modulators (MZMs) in our study, with *c* being the index of the channel $\lambda _c$ at which the MZM is centered. We assume that MZMs have voltage-controlled Phase Shifters (PS) in both arms (indexed as “1/2” for upper/lower arm, respectively) and are operated in push-pull configuration with DC induced phase shifts given as $\phi _{\mathrm {DC},1/2} = 2\pi n(V_{\mathrm {DC},1/2}, \lambda ) L_\mathrm {DC}/\lambda$ and RF induced as $\phi _{1/2}(\pm V_\mathrm {RF}, \lambda ) = \phi _0 (\lambda ) \pm \Delta \phi (V_\mathrm {RF}, \lambda )$ with $\phi _0 = 2\pi n_0(\lambda ) L /\lambda$ and $\Delta \phi = 2\pi \Delta n(V_\mathrm {RF}, \lambda ) L /\lambda$ where *L* and $L_\mathrm {DC}$ denote the lengths of RF and DC active regions and $n = n_0 + \Delta n$, with $n_0$ and $\Delta n$ being the refractive index at zero applied voltage and its deviation when the voltage is applied. The transfer function of the MZM is given as

5 $$\begin{aligned} t_\mathrm {MZM} (\lambda ) = \cos \left\{ \left[ 2 \Delta \phi (\lambda ) + \phi _{\mathrm {DC},1} (\lambda ) - \phi _{\mathrm {DC},2} (\lambda ) \right] /2 \right\} \times \exp \left\{ i \left[ 2 \phi _0 (\lambda ) + \phi _{\mathrm {DC},1} (\lambda ) + \phi _{\mathrm {DC},2} (\lambda ) \right] /2 \right\} \, , \end{aligned}$$

and is tailored such that $t_\mathrm {MZM} (\lambda _c) = x_{n,c}$ by choosing the DC voltages (biases) which induce phase shifts separated by $\pi$, implying $\phi _{\mathrm {DC},1} = \phi _\mathrm {DC} - \pi$ and $\phi _{\mathrm {DC},2} = \phi _\mathrm {DC}$. Assuming that the modulation-induced phase-variation does not contribute significantly to the overall wavelength dependence, the MZM transfer function can be approximated by

6 $$\begin{aligned} t_\mathrm {MZM} (\lambda ) \approx \sin \Delta \phi (\lambda _c) \exp \left\{ i \left[ \phi _0 (\lambda ) + \phi _\mathrm {DC}( \lambda ) - \frac{\pi }{2} \right] \right\} \, . \end{aligned}$$

For modes of operation #3 and #4, MZM transfer function will be centered at a certain $\lambda _c$, i.e., optimized to deliver targeted input $x_{n,c}$ at the given channel by enforcing $\Delta \phi (V_\mathrm {RF}, \lambda _c) = \arcsin x_{n,c}$ and setting the argument of the exponential function in Eq. (5) to a multiple of $2\pi$. For any other channel *m*, the imprinted value $x_{n,m,c}$ will deviate from the targeted one. Following the detailed analysis of the input modulator operation given in Section 5 of Supplementary Document, relying on the 1$\mathrm{{st}}$ order Taylor expansion of the phases $\phi _0 (\lambda )$ and $\phi _\mathrm {DC} (\lambda )$ around $\lambda _c$, we find that the *m*th channel of the *n*th axon carries the input value given by

7a $$\begin{aligned} x_{n,m,c}&\approx x_{n,c} \exp \left( -i \xi _{m,c}^{(x)} \right) \, , \end{aligned}$$

7b $$\begin{aligned} \xi _{m,c}^{(x)}&= 2 \left( p_x + q_x + \frac{1}{4} \right) \pi \frac{ n_\mathrm {g} (\lambda _c) }{ n(\lambda _c) } \frac{1}{\lambda _c} (m - c) \Delta \lambda _1 \, , \end{aligned}$$

where $p_x = n_0(\lambda _c) L/\lambda _c$ and $q_x = n(V_\mathrm {DC},\lambda _c) L_\mathrm {DC}/\lambda _c$ stand for normalized lengths of RF and DC phase shifters within the MZM and are restricted to $p_x, q_x \in {\mathbb {N}}$, $n_\mathrm {g}$ is the group refractive index, and $\Delta \lambda _1 = \lambda _{m+1} - \lambda _m$ denotes channel spacing (assuming equidistant channels). Parameter $\xi _{m,c}^{(x)}$ represents the phase shift accumulated by channel *m* and reveals four important facts: (i) it does not depend on targeted $x_{n,c}$ value implying that the phase accumulation does not vary with the input sequence; (ii) it does not depend on the axon index *n*, implying that all axons introduce the same amount of phase accumulation that can be compensated outside the OLAU rather than within the OLAU itself; (iii) it depends on the difference between *m* and *c* implying that all side channels of the same order have the same phase accumulation which magnitude increases with $|m-c|$; (iv) it increases with the channel spacing $\Delta \lambda _1$.

In order to implement the weights $w_{n,c}$ a combination of MZM and an independent PS can be used. Depending on targeted application, amplitude modulation can be achieved either through absorption control^4,8,23^ or by employing interferometric modules^9,10,22^ using either T/O or E/O PSs. Aligning with the majority of reported state-of-the-art coherent layouts targeting inference, and thus allowing slow reconfiguration rates, we choose thermally controlled PSs both within MZM’s arms and in the PS that follows. Here we note that cointegration of the E/O (input) and T/O (weight) modulators requires careful planning in order to avoid thermal crosstalk but has turned into a well-established process during the last years, with significant on-chip demonstrations of co-integrated E/O and T/O structures both in the fields of silicon-based transceivers^25^, as well as in neuromorphic photonics^22,23^. Adopting thermally insulating trenches and/or heat shunts^26,27^ or more elaborate approaches such as thermal eigenmode decomposition^28^, can be additionally employed, if necessary, in order to ensure reliable operation of both device types in diverse PIC platforms, including Si and InP ones. Unlike E/O MZM, the T/O MZM cannot be operated in push-pull configuration; instead, it can be made asymmetrical by changing the length of the waveguide(s) in one or both of its arms to achieve a built-in phase difference of $2 \theta$ at the nominal temperature $T_0$ and $\lambda _c$, or, in other words, it will be biased at $2\theta$-point. At any point in time, only one PS is being used for adjusting the weight magnitude depending on the ratio of $|w_{n,c}|$ and $\cos \theta$. This is reflected in the electric field transfer function of the MZM-PS system

8 $$\begin{aligned} t_\mathrm {MZM-PS} ( \lambda ) = \cos \left[ \theta - \mathrm {sgn} \left( |w_{n,c}| - \cos \theta \right) \Delta \phi (\Delta T, \lambda )/2 \right] \times \exp \left\{ i \left[ \phi (T_0, \lambda ) + \Delta \phi (\Delta T, \lambda )/2 + \phi _3(\lambda ) \right] \right\} \, , \end{aligned}$$

where $\phi (T_0, \lambda ) = 2\pi n (T_0, \lambda ) L/\lambda$ is the phase accumulated in MZM at $T_0$, $\Delta \phi (\Delta T, \lambda ) = 2\pi \Delta n (\Delta T, \lambda ) L/\lambda$ is the phase shift due to applied differential temperature $\Delta T$, and $\phi _3 (T, \lambda ) = 2\pi n (T, \lambda ) L_3/\lambda$ is the phase accumulated in the standalone PS. Similar to the case of input MZM, we can neglect the contribution of $\Delta \phi$ variation with the wavelength and approximate the MZM-PS transfer function by

9 $$\begin{aligned} t_\mathrm {MZM-PS} ( \lambda ) \approx |w_{n,c}| \times \exp \left\{ i \left[ \phi (T_0, \lambda ) + \Delta \phi (\Delta T, \lambda _c)/2 + \phi _3(\lambda ) \right] \right\} \, , \end{aligned}$$

taking into account that it will be centered at $\lambda _c$ yielding $t_\mathrm {MZM-PS} ( \lambda _c ) = w_{n,c}$, implying also $\phi (T_0, \lambda _c) = 2 p_w \pi$ and

10a $$\begin{aligned} \Delta \phi (\Delta T, \lambda _c)&= 2 \, \mathrm {sgn} \left( |w_{n,c}| - \cos \theta \right) \left( \theta - \arccos |w_{n,c}| \right) \, , \end{aligned}$$

10b $$\begin{aligned} \phi _3 (\lambda _c)&= \frac{1 - \mathrm {sgn} (w_{n,c}) }{2} \pi + 2 p_s \pi - \frac{1}{2} \Delta \phi (\Delta T, \lambda _c) \, , \end{aligned}$$

where $p_w, p_s \in {\mathbb {N}}$. For any channel $m \ne c$, staying restricted to the 1st order approximation and assuming $p_w, p_s \gg 1$ which is expected in all cases of practical interest, following the detailed derivation given in Section 6 of Supplementary Document, we find that the *m*th channel of the *n*th axon carries the weight

11a $$\begin{aligned} w_{n,m,c}&\approx w_{n,c} \exp \left( -i \xi _{m,c}^{(w)} \right) \, , \end{aligned}$$

11b $$\begin{aligned} \xi _{m,c}^{(w)}&= 2 ( p_w + p_s ) \pi \frac{ n_\mathrm {g} (\lambda _c) }{ n(\lambda _c) } \frac{1}{\lambda _c} (m - c) \Delta \lambda _1 \, , \end{aligned}$$

where $p_w = n(T_0,\lambda _c) L/\lambda _c$ and $p_s = n(T_0,\lambda _c) L_3/\lambda _c$ represent normalized lengths of the PSs within the MZM and the standalone PS, respectively, with *L* and $L_3$ being their lengths. Same conclusions enlisted earlier for $\xi _{m,c}^{(x)}$ hold for $\xi _{m,c}^{(w)}$.

For signal multiplexing and demultiplexing Arrayed Waveguide Gratings (AWGs) are used, with a flat channel-wise spectral response over the frequency band of interest. We assume that the AWG’s power transfer function is given as a parabola in logarithmic domain, symmetrical and centered at the channel’s wavelength, and that it introduces negligible overall losses. In linear domain, the transfer function corresponds to the far-field shape, i.e., a Gaussian function versus the wavelength^29^. The crosstalk of the AWG, defined as the ratio of powers of the first suppressed channel and the pass channel, is denoted as $r_\mathrm {AWG}$ in linear terms, or $R_\mathrm {AWG}$ in logarithmic (dB) domain. In what follows, we assume zero insertion loss (IL) and restrict ourselves to the 1$\mathrm{{st}}$ order approximation where it is assumed that the crosstalk is relevant only between adjacent channels. We also assume that the curvature of the output free-propagating region of the AWG matches the curvature of the Gaussian field (its equiphase line in transversal plane) yielding zero-phase difference between adjacent output waveguides.

When passing through the DEMUX, channel *m* will be distributed not only to the *m*th output port, but also to ports $(m \pm 1)$, with the ratio of powers being determined by $r_\mathrm {AWG}$. This will cause the *m*th channel in adjacent waveguides to be modulated by input or weight targeted at channels $(m \pm 1)$. Subsequently, when collected by MUX, reversed process will follow, which will gather all the signals back to the output, leading to mixing of inputs or weights belonging to the three adjacent paths, with the appropriate coefficients. Following the detailed derivation given in Section 7 of Supplementary Document, we find that the actual, imprinted value of the input in modes of operation #1 and #2 deviates from the targeted one as

12 $$\begin{aligned} x_{n,m}^\mathrm {AWG} \approx x_{n,m} + r_\mathrm {AWG} (x_{n,m-1} - 2 x_{n,m} + x_{n,m+1}) \, , \end{aligned}$$

under the constrain $x_{n,0} = x_{n,M+1} = 0$ and with the same formalism being applied to weights in modes #1 and #3, and biases in all modes of operation. Unlike the deviation coming from using a single modulator for multiple channels, which can be compensated to a certain extent, the crosstalk originating from the AWG cannot be easily counteracted outside the OLAU as it its pattern-dependent and, consequently, depends both on the index of the axon *n* and index of the channel *m*.

Having identified wavelength-dependent behaviour of the PPNN’s constituent components, its experimental diagonal transfer matrix, $Q_\mathrm {e}$, can be derived based on the PPNN configuration for different modes of operation, as per Tables 1 and 2, following the path of the signal in Fig. 1e, relying on Eq. (12) for modeling the AWG response, and Eqs. (5) and (8) for unapproximated input and weight modulator transfer functions. Similar as in the case of $Q_\mathrm {t}$ in Eq. (2a), we disregard the accumulated phase shift in $Q_\mathrm {e}$ and restrain our focus only to the phase difference between the bias branch and the OLAU and between the axons in the OLAU itself, as these lead to potential performance deterioration through impairment of interference conditions. In order to perform phase alignment between the bias branch and the OLAU in modes of operation which assume using a single modulator for enforcing inputs or weights to multiple channels (mode #3 for inputs and #2 for weights), we modify the bias branch transfer matrix from ${\widetilde{W}}_\mathrm {b}$ to ${\widetilde{W}}_\mathrm {b} \Xi _c^{(w)}$ in mode #2 or ${\widetilde{W}}_\mathrm {b} \Xi _c^{(x)}$ in mode #3, where

13 $$\begin{aligned} \Xi _c^{(x/w)} = \mathrm {diag} \left[ \exp \left( i \xi _{1,c}^{(x/w)} \right) , \ldots , \exp \left( i \xi _{M,c}^{(x/w)} \right) \right] \, , \end{aligned}$$

with $\xi _{m,c}^{(x)}$ and $\xi _{m,c}^{(w)}$ being defined by Eqs. (7b) and (11b), respectively. In this manner, channel-selective phase accumulation originating from Eqs. (7a) and (11a) is cancelled, as detailed in Section 8 of Supplementary Document. It should be stressed that $Q_\mathrm {e}$ derived based on Eqs. (7), (11) and (12) is approximate and, even though the phase compensation is carried out via the PSs in the bias branch, certain deviation from $Q_\mathrm {t}$ will remain. In the forthcoming analysis, these will be quantified by absolute, $\Delta q_m = q_{\mathrm {e},m} - q_{\mathrm {t},m}$, and relative error, $\delta q_m = |\Delta q_m|/q_{\mathrm {t},m}$, between the experimental, $q_{\mathrm {e},m}$, and targeted, $q_{\mathrm {t},m}$, diagonal matrix elements. The errors can be derived based on the expressions correlating $q_{\mathrm {e},m}$ and $q_{\mathrm {t},m}$ in Section 8 of Supplementary Document.

## PPNN performance analysis

For our case-study, we assume silicon platform, with the refractive index dependence on wavelength at different temperatures taken from^30^. At $\lambda _c = 1.55 \, \mu \mathrm {m}$ and $T_0 = 293 \, \mathrm {K}$ we have $n = 3.4757$ and $n_\mathrm {g} = 3.5997$. In case of E/O modulators, unless doping is severe and/or composite materials are used, optical properties of the undoped silicon (where the majority of light is confined) remain the same as above, whereas the dependence of the refractive index on the voltage is assumed to be approximately linear for the voltage ranges of interest.

Using Monte-Carlo method, we observe $10^4$ sets of random, uniformly distributed input and weight values, chosen on the domain $x_{n,m} \in [0,1]$ and $w_{n,m} \in [-1,1]$ and keep the bias fixed to ${\widetilde{w}}_{\mathrm {b},m} = 1$ in order to ensure that the information about the sign of the sum is preserved when transitioning to the power domain. When employing PPNN in trained environment, bias weight can take any value from ${\widetilde{w}}_{\mathrm {b},m} \in [-1,1]$ imposed by the training algorithm. Following the simulation, the diagonal matrix elements $q_{\mathrm {t},m}$ and $q_{\mathrm {e},m}$ are aggregated and 2-D scatter plots analyzed using multivariate statistical approach to determine deviations in terms of absolute and relative error.

**Figure 4:**
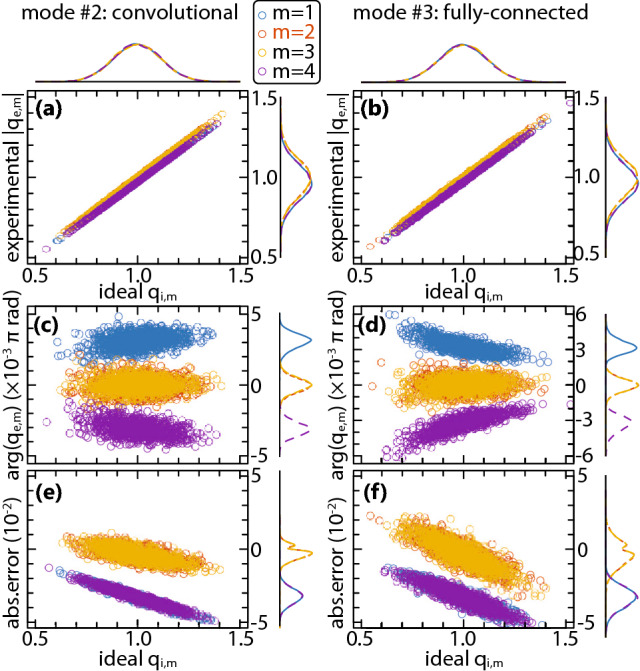
Comparison between the convolutional (#2, left-hand-side) and the fully-connected (#3, right-hand-side) mode of PPNN operation with $M=4$ channels, optimized for operation at channel $c=2$, and $N=8$ axons for $\Delta \lambda _1 = 0.8 \, \mathrm {nm}$ and $R_\mathrm {AWG} = -15 \, \mathrm {dB}$. Channel-wise color coded 2-D scatter plots of the targeted matrix element $q_{\mathrm {t},m}$ and (**a**), (**b**) the magnitude and (**c**), (**d**) the argument of the experimental matrix element $|q_{\mathrm {e},m}|$ and (**e**), (**f**) the algebraic magnitude of the absolute deviation of the experimental from targeted matrix element, $\mathrm {sign}( {\mathfrak {R}}{\mathfrak {e}} \{ \Delta q_m \} ) |\Delta q_m|$, with $\Delta q_m = q_{\mathrm {e},m} - q_{\mathrm {t},m}$, all with displayed univariate kernel probability density plots on the corresponding horizontal and vertical axes of the scatter plots.

Figure 4 shows 2-D scatter plots for two different modes of operation, convolutional (left-hand-side) and FC (right-hand-side), for T/O MZM biasing point $\theta = \pi /3$, normalized lengths $p_x = q_x = 100$ and $p_w = p_s = 50$, nominal channel spacing $\Delta \lambda _1 = 0.8 \, \mathrm {nm}$, translating to approximately $100 \, \mathrm {GHz}$ in frequency domain, and $R_\mathrm {AWG} = -15 \, \mathrm {dB}$. Phase alignment between the bias branch and the OLAUs output has been carried out following Eq. (13).

In terms of magnitude of the experimental matrix element, $|q_{\mathrm {e},m}|$, versus the targeted matrix element, $q_{\mathrm {t},m}$, both modes of operation show similar performance, as confirmed by Fig. 4a, b, when optimized for the same channel, $c = 2$, out of $M=4$ color-coded channels in the PPNN when a single modulator is used, or, optimized for *m* if a modulator per channel is used. The Spearman’s rank correlation coefficient $\rho$ in both cases given in Fig. 4a, b exceeds 0.999 for all 4 observed channels, indicating almost perfect monotonic relation between the two quantities. The univariate Probability Density Functions (PDFs) of both $q_{\mathrm {t},m}$ and $|q_{\mathrm {e},m}|$ retain Gaussian shape, complying with Central Limit Theorem (CLT). Nevertheless, a slight downshift in the means of edge channels’ PDFs can be observed ($m=1$ and $m=4$), or, in other words, reduction in the mean value of the experimental matrix element comparing to the targeted one. The downshift implies that edge channels encounter greater power loss than the inner ones during the propagation through PPNN, which can be attributed to the DEMUX/MUX pairs embracing the modulators in the input and weight banks. Namely, as the edge channel gets demultiplexed, the fraction of its optical power that is proportional to the crosstalk strength ($r_\mathrm {AWG}$) and is sent to an adjacent channel not supported by PPNN (channel 0 for $m=1$ and channel $M+1$ for $m = M$) gets irreversibly lost during demultiplexing step. This effect is not observed for inner channels, since they distribute their crosstalk signals to the adjacent channels which are supported by PPNN, and can be later on collected by MUX, as described in Section 7 of Supplementary Document. This edge-channel loss penalty is captured by $x_{n,0} = x_{n,M+1} = 0$ and $w_{n,0} = w_{n,M+1} = 0$ in Eq. (12) and its counterpart for $w_{n,m}^\mathrm {AWG}$.

Scatter plots of the argument of $q_{\mathrm {e},m}$ versus $q_{\mathrm {t},m}$, given in Fig. 4c, d, reveal that phase alignment based on the approximate expression given by Eqs. (7b) and (11b) yields excellent results, bringing the residual phase shifts below $0.01\pi \, \mathrm {rad}$. The distribution of $\mathrm {arg}(q_{\mathrm {e},m})$ is well approximated by Gaussian owing to CLT and depends to a certain extent on the targeted matrix element $q_{\mathrm {t},m}$ value. It can be also noticed that the edge channels ($m=1$ and $m=4$) suffer a shift of the PDFs as was the case with the PDFs describing the magnitude of $q_{\mathrm {e},m}$, arising from non-symmetrical phase shifts seen by the 1$\mathrm{{st}}$ and $M\mathrm{{th}}$ channel. This time, however, the shift of the mean is of different sign: positive for the 1$\mathrm{{st}}$ and negative for the $M\mathrm{{th}}$ channel. In both cases, the shift originates from the crosstalk in the bias branch, where phase compensation is performed. Looking at the bias counterpart of (12), the crosstalk term is proportional to $r_\mathrm {AWG} ( {\widetilde{w}}_{\mathrm {b},m-1} - 2 {\widetilde{w}}_{\mathrm {b},m} + {\widetilde{w}}_{\mathrm {b},m+1} )$, and, having ${\widetilde{w}}_{\mathrm {b},m} = 1$ for all supported channels $m \in [1,M]$, should amount to 0. Yet, when $m = 1$ or $m = M$, the signals are not counterbalanced since ${\widetilde{w}}_{\mathrm {b},0} = {\widetilde{w}}_{\mathrm {b},M+1} = 0$, leaving a residual crosstalk term proportional to $-r_\mathrm {AWG}$, which is multiplied by $\Xi _c^{(x)}$ or $\Xi _c^{(w)}$ depending on the mode of operation, as detailed in Section 8 of Supplementary Document. On the other hand, the elements of $\Xi _c^{(x/w)}$ depend on the difference between the observed channel *m* and the channel with respect to which the modulator was centered, *c*, as (7b) and (11b) show. This leads to phase shifts of different signs for the 1$\mathrm{{st}}$ and the $M\mathrm{{st}}$ channel, since the typical choice is $c = \lceil M/2 \rceil$. Regardless of means being shifted, standard deviations of the corresponding quasi-Gaussian PDFs remain similar as for the inner channels ($m = 2$ and $m = 3$).

Finally, in Fig. 4e, f, we observe the algebraic magnitude of the absolute error between the experimental and the targeted transfer matrix elements, $\mathrm {sign}( {\mathfrak {R}}{\mathfrak {e}} \{ \Delta q_m \} ) |\Delta q_m|$. The effect of mean drifting for edge channels, observed in Fig. 4a, b, can now be quantified and, for all analyzed cases stays below $|\Delta q_m| < 0.06$ which yields the maximum relative error of the order of $4 \%$ for edge channels. In case of inner channels, the error is centered in the proximity of 0 and, for a given $\Delta \lambda _1$ and $R_\mathrm {AWG}$ stays below $2 \%$ in $> 90\%$ of analyzed random sets.

**Figure 5:**
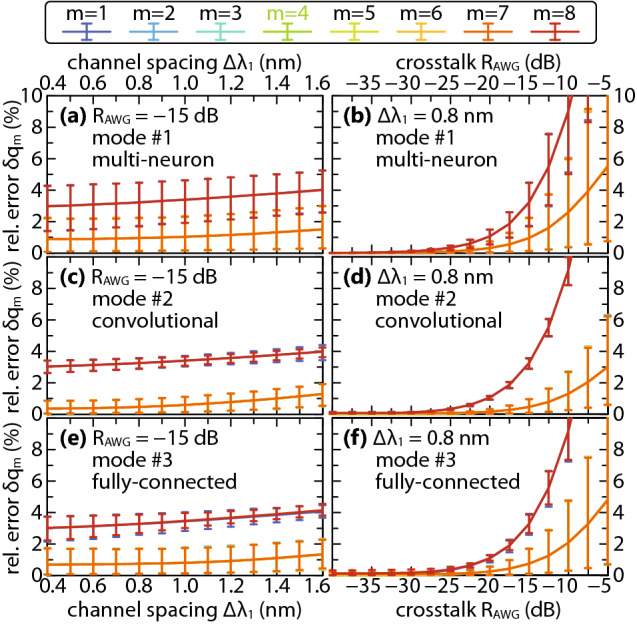
Mean relative errors of the matrix element $\delta q_m$ (given in percent) with $5 \%$ to $95 \%$ confidence bounds for (**a**), (**b**) multi-neuron, (**c**), (**d**) convolutional, and (**e**), (**f**) FC mode of operation, depending on (**a**), (**c**), (**e**) channel spacing for $R_\mathrm {AWG} = -15 \, \mathrm {dB}$ and (**b**), (**d**), (**f**) AWG crosstalk for $\Delta \lambda _1 = 0.8 \, \mathrm {nm}$.

We extend our analysis to all multichannel modes of PPNN operation according to Table 1 for $\Delta \lambda _1$ from 0.4 to $1.6 \, \mathrm {nm}$ (translating to grid spacing of 50–$200 \, \mathrm {GHz}$) and $R_\mathrm {AWG}$ from $-\,40$ to $-\,5 \, \mathrm {dB}$, accounting for $M=8$ channels centered at $c = 4$ when a single modulator for all channels is used, and at *m* otherwise, aiming to determine the influence of various system parameters on the relative error of the matrix element, $\delta q_m$. Figure 5 shows mean values of relative errors over the collection of $10^4$ analyzed samples, together with 5–95% confidence bounds versus $\Delta \lambda _1$ for AWG crosstalk of $-15 \, \mathrm {dB}$ and versus $R_\mathrm {AWG}$ for channel spacing of $0.8 \, \mathrm {nm}$. As observed in scatter plots given in Fig. 4, we again confirm based on Fig. 5 that edge channels ($m=1$ and $m=8$) introduce similar amount of error (lines are overlapping), which is greater than the error encountered by inner channels ($2 \le m \le 7$), also overlapping among themselves. The underlying cause is related to the asymmetry in the filed magnitude and phase shifts accumulated by edge channels when passing through AWG, as previously elaborated. The important conclusion stemming from this overlap is that the number of employed channels *M* does not pose a challenge for any of the PPNN modes of operation, as long as phase compensation is done within the bias branch following Eq. (13).

Comparing different modes of operation in Fig. 5 reveals that the mean relative error, be it higher for the edge channels or lower for the inner ones, remains fairly similar for different modes of operation (excluding very high $R_\mathrm {AWG}$), having weaker dependence on $\Delta \lambda _1$ than on $R_\mathrm {AWG}$. For $R_\mathrm {AWG} = -15 \, \mathrm {dB}$ it does not exceed $4 \%$ for any analyzed $\Delta \lambda _1$, however, as the crosstalk increases, the mean error shoots up exponentially, surpassing $10 \%$ for the edge channels at $R_\mathrm {AWG} = -10 \, \mathrm {dB}$ and remaining within manageable values of up to $6\%$ for the inner ones even at $R_\mathrm {AWG} = -5 \, \mathrm {dB}$. On the other hand, there is a significant difference in the confidence interval between the modes of operation: it is widest for the multi-neuron mode of operation, given in Fig. 5a, b, and reduces for convolutional and FC modes, given in Fig. 5c–f, implying that, although not common, large errors can occur in multi-neuron case. Same evolution of the confidence interval can be seen with respect to AWG crosstalk, Fig. 5b, d, f, revealing that having more DE/MUX stages in mode #1 comparing to the remaining 2 modes of operation is actually responsible for its sizeable spread of errors, as is expected based on the Eq. (12).

Looking at convolutional, Fig. 5c, d, and FC mode of operation, Fig. 5e, f, difference can be observed in the confidence intervals, and to a certain extent in the mean relative error for the inner channels, indicating that convolutional mode of operation seems to exhibit better overall performance. Yet, from architectural point of view, Figs. 1, 2 and 3, the two are nearly interchangeable. At the same time, our analysis shows that the normalized modulator lengths $p_x$, $q_x$, $p_w$ and $p_s$ play marginal role in relative error means and confidence intervals, as was expected having in mind that the accumulated phase given by Eqs. (7b) and (11b) is compensated by the PSs within the bias modulator bank following Eq. (13). The difference, thus, comes in response to different domains of inputs and weights, i.e., the the quantities enforced jointly to all-channels and the ones enforced on per-channel bases. Repeating the analysis from Fig. 5 for weights restricted to the same domain as inputs, namely $w_{n,m} \in [0,1]$, confirms that the confidence intervals slightly reduce for both modes of operation and, more importantly, become similar in magnitude. This can be explained by reducing the magnitude of crosstalk in weight modulator bank in the FC mode of operation by halving the range of the values $w_{n, m \pm 1}$ can take in the equivalent of Eq. (12) for $w_{n,m}^\mathrm {AWG}$.

**Figure 6:**
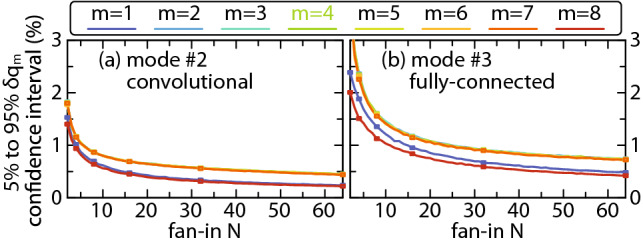
Relative error 5–95% confidence interval (given in %) versus the neuron fan-in *N* at $\Delta \lambda _1 = 0.8 \, \mathrm {nm}$ and $R_\mathrm {AWG} = -15 \, \mathrm {dB}$ for (**a**) convolutional and (**b**) fully-connected mode.

The study of the PPNN performance on fan-in has been carried out for *N* ranging from 2 to 64 and reported in Fig. 6 for convolutional and FC configuration. A clear trend can be observed for both modes of operation where the confidence interval reduces with the increase of *N*, stemming from narrowing of the univariate PDF of both $q_{\mathrm {t},m}$ and $|q_{\mathrm {e},m}|$, complying with CLT, whereby the standard deviation decreases with $1/\sqrt{N}$. The values of the mean relative error remain similar to the ones in Fig. 5 across different *N* values, implying that, similar to other analyzed parameters, the number of axons does not pose a challenge to PPNN operation.

## Implementation considerations and perspectives

Here we discuss the practical aspects of PPNN implementation, focusing on insertion losses ($\mathrm {IL}_\mathrm {PPNN}$), power consumption ($P_{\mathrm {PPNN},m}$), footprint ($A_{\mathrm {PPNN},m}$) and throughput ($T_{\mathrm {PPNN},m}$), jointly shaping the energy- and footprint-efficiency, defined as the ratio of the throughput and the power consumption or the PPNN area, respectively. We recognize the penalties introduced by sub-optimal resource employment, such as powering off some of the LDs or keeping some of the axons dark, i.e., using less channels ($M_A \le M$) or less axons ($N_A \le N$) than the PPNN supports. Based on the detailed study reported in Section 9 of Supplementary Document, we find the respective values per number of active channels for power-of-2 splitting and combining stages

14a $$\begin{aligned} \mathrm {IL}_\mathrm {PPNN}&= 2( 1 + \mathrm {S}_\mathrm {X} + \mathrm {S}_\mathrm {O} ) \mathrm {IL}_\mathrm {MUX} + 3 \mathrm {IL}_\mathrm {S} + 2( 1 + \log _2 N ) \mathrm {IL}_\mathrm {C} + \mathrm {IL}_\mathrm {X} + \mathrm {IL}_\mathrm {W} + \mathrm {IL}_\mathrm {R}^\mathrm {(A)} + 10 \log _{10} (N/N_A) \, , \end{aligned}$$

14b $$\begin{aligned} P_{\mathrm {PPNN},m}&= \ \frac{P_\mathrm {LD}}{\eta _\mathrm {wp}} + \ \left( \frac{1 - \mathrm {S}_\mathrm {X}}{M_A} + \mathrm {S}_\mathrm {X} \right) \times \left( N P_\mathrm {X}^\mathrm {(DC)} + N_A P_\mathrm {X}^\mathrm {(RF)} \right) + \left[ N_A \left( \frac{1 - \mathrm {S}_\mathrm {O}}{M_A} + \mathrm {S}_\mathrm {O} \right) + 1 \right] \times P_\mathrm {W} \nonumber \\&\quad + \ \frac{N}{M_A} \sum _{i = \{\mathrm {X,W,O}\}} \mathrm {S}_i P_\mathrm {S} \, , \end{aligned}$$

14c $$\begin{aligned} A_{\mathrm {PPNN},m}&= \left[ 2 \left( 1 + \log _2 N \right) L_\mathrm {C} + L_\mathrm {A} \right] \times \left[ \frac{M}{M_A} (N + 1) + \frac{1}{M_A}(N - 1) \right] L_\Delta \, , \end{aligned}$$

14d $$\begin{aligned} T_{\mathrm {PPNN},m}&= N_\mathrm {A} B_\mathrm {X} \, , \end{aligned}$$

where $\mathrm {IL}_i$, $L_i$ and $P_i$ denote per-device insertion losses, length and power consumption, with the exception of $P_\mathrm {LD}$ which stands for the optical power of the LD per channel. Indices $i\in \mathrm {\{MUX,S,C,X,W,R\}}$ refer, in the given order, to DE/MUX, switch, X-coupler, input amplitude modulator, weight amplitude and phase modulator and routing waveguides. Moreover, $\eta _\mathrm {wp}$ is the wall-plug efficiency of the LD, $L_\mathrm {A}$ is the total length of an axon, $L_\Delta$ distance between lateral waveguides, $B_\mathrm {X}$ is the datarate of the input modulator, and $\mathrm {S}_\mathrm {\{X,W,O\}}$ are the switch states defined in Table 1 depending on the mode of operation.

The first two terms of $\mathrm {IL}_\mathrm {PPNN}$ in (14a) denote the penalty introduced by multichannel operation ($\sim \mathrm {IL}_\mathrm {MUX}$) and programmability ($\sim \mathrm {IL}_\mathrm {S}$), whereas the last term denotes the penalty in the form of irreversibly lost optical power when $N_A < N$ axons are used. No IL penalty is observed when $M_A < M$ channels are employed.

The PPNN power consumption per channel, given by (14b), is governed by all of its active components, which are, in turn, powered on based on the states of the switches and modes of operation. The power consumption of the optional Transimpedance Amplifier (TIA) and Temperature Controller (TEC) are excluded from the analysis as they would contribute to the total power consumption in a similar manner regardless of the multichannel operation or PNN programmability. Comparing to its predecessor, dual-IQ coherent linear neuron^21^, power consumption of PPNN in modes #1 and #4 is similar to that of dual-IQ, with a minor penalty $\sim P_\mathrm {S}$ in PPNN case owing to its programmability. However, operating in either mode #2 (convolutional) or #3 (fully-connected) brings power savings in PPNN case through weight (#2) or input (#3) modulator sharing, since the coefficients pondering $P_\mathrm {W}$ and $P_\mathrm {X}^\mathrm {(DC)/(RF)}$, respectively, get divided by the number of active channels, $M_A$, implying increased energy-efficiency of the PPNN comparing to using $M_A$ dual-IQ neurons.

Comparing the PPNN footprint per channel, given by (14c), to that of dual-IQ, we can observe both longitudinal and lateral penalty, the former due to DE/MUXes and switches making $L_\mathrm {A}$ longer for PPNN than for dual-IQ, and the latter due to the existence of two alternative routes a signal can take within the input and/or weight banks. Focusing on two corner scenarios, when (i) $M_A = M \sim N$ and (ii) $M_A = 1$, the lateral footprint penalty due to multichannel operation and programmability ranges from multiplicative factors of (i) $\sim (1 + 2/N)$ (best-case scenario) to (ii) $M (1 + 1/N) + 1 - 1/N$ (worst-case scenario). The second case reveals that power-saving mode of operation comes at a price of footprint penalty proportional to the number of channels for which PPNN was designed.

The thorough study on wavelength dependence of individual components could be further extended to incorporate the temperature dependent operation of devices and statistical differences between the employed components. Temperature dependent operation would provide useful information regarding the performance reliability in realistic conditions where on-chip temperatures up to 80–100$^\circ$C can be encountered. An extended analysis where statistical differences between the employed components are taken into account would provide a clearer insight with respect to its practical perspectives, since current silicon photonic platforms don’t guarantee identical performance for identical devices, calling for a system tolerance analysis. The study can also be expanded to different types of input/weight modulators which are governed by different amplitude and phase equations, aiming to conclude to analytical expressions for deviation compensation.

On the system level, two upscaling directions can be taken. One relates to interconnection of multiple PPNNs and employing them in inference task in order to estimate their accuracy under a non-random load. The second relies on the positive impact that the increase of number of axons has on the reduction of the confidence interval of relative error reported in Fig. 6. This indicates that PPNN architecture can be reliably extended into a two-dimensional arrangement, similar to our recently proposed photonic crossbar^31^, yielding *K* spatially separated neuron outputs. Boosted by WDM, crossbar could support a total of $K \times M$ logical outputs, while also offering flexibility to switch between the different modes of operation, approaching to the photonic FPGA concept.

## Conclusion

In this manuscript we present an in-situ reconfigurable coherent PNN, exploiting the wavelength domain for achieving parallel operation of multiple neurons with flexible, user-defined interconnection graph, supporting four distinct modes of operation, among others convolutional and fully-connected layer. We carry out a detailed analytical study of the modulator and DE/MUX wavelength dependence, offering a simple approach for restoring the PNN fidelity through phase alignment of the bias signal, revealing that the majority of the residual errors comes from the crosstalk in DE/MUX stages. The analytical approach is benchmarked against Monte-Carlo simulation showing that the residual relative error typically remains within the manageable 2% range for AWG crosstalk of up to $-20 \, \mathrm {dB}$. More importantly, the PNN performance does not degrade with the increase of number of channels or the neuron fan-in as long as phase alignment in the bias branch is carried out, supporting seamless network upscaling, including the extension to multi-column arrangements for vector-by-matrix multiplication. The relative error dependence on channel spacing is weak, allowing the PNN to be operated equally well in coarse and dense WDM systems.

## Supplementary Information

Supplementary Information.

## Data Availability

The datasets generated during and/or analysed during the current study are available from the corresponding author on reasonable request.
