# Supplementary material for: Programmable photonic neural networks combining WDM with coherent linear optics
Source: Sci Rep. 2022 Apr 4;12:5605. doi: 10.1038/s41598-022-09370-y (PMC8980092; doi:10.1038/s41598-022-09370-y)
Supplement: Supplementary file 1 — Supplementary Information. [file 41598_2022_9370_MOESM1_ESM.pdf]

Programmable Photonic Neural Networks Combining WDM with Coherent Linear Optics: Supplementary Document

Angelina Totovic[1,*], George Giamougiannis[1], Apostolos Tsakyridis[1], David Lazovsky[2], and Nikos Pleros[1]

[1]Department of Informatics, Center for Interdisciplinary Research and Innovation - CIRI, Aristotle University of Thessaloniki, Balkan Center - Building A, 10th Km Thessalonikis-Thermis Av, 57001, Thessaloniki, Greece
[2]Celestial AI, 100 Mathilda Pl. Suite 170, Sunnyvale, CA 94086, USA
[*]angelina@auth.gr

ABSTRACT

This document provides supplementary information to "Programmable photonic neural networks combining WDM with coherent linear optics", giving the detailed information about PPNN modes of operation and the theoretical foundations, including a detailed study of the wavelength-dependent operation of the splitters, combiners, switches and input and weight amplitude and phase modulators employed in PPNN, concluding with the study of performance metrics focusing on insertion losses, power consumption and footprint and the related penalties arising from multichannel, programmable operation.

S1 PPNN modes of operation

Depending on the configuration of the switches in Photonic Neural Network (PNN) axons, introduced in main body of the Manuscript, Fig. 1, the Programmable (P)-PNN can operate in 4 distinct modes illustrated in Fig. S1. Left-hand side of the Fig. S1 shows the n-th branch (axon) of the PPNN, according to Fig. 1(e) from the main body of the Manuscript, with inaccessible (inactive) optical paths represented as semi-transparent, whereas the right-hand side of Fig. S1 shows the corresponding abstraction of the NN layer.

When the switch $S_{\mathrm{X},n}$ is in its `on` or `up` state ($S_{\mathrm{X},n} = 1, \forall n$), as in Fig. S1(a), (b), each channel m carries its designated input sequence $X_m = [x_{1,m}, \ldots, x_{N,m}]$, depicted by appropriately colored input circles in the right-hand side abstractions, otherwise, when $S_{\mathrm{X},n}$ is in its `off` or `down` state ($S_{\mathrm{X},n} = 0, \forall n$), as in Fig. S1(c), (d), all channels $m \in [1, M]$ carry identical input sequence $X_0 = [x_{1,0}, \ldots, x_{N,0}]$, represented by grey circles in the right-hand side abstractions.

Similar conclusion can be made for weights, with the exception that they are controlled by a combination of input, $S_{\mathrm{X},n}$, and weight switches, $S_{\mathrm{W},n}$. Let us introduce a convention where the bar (straight) position of the weight switch is assumed as its `on` state ($S_{\mathrm{W},n} = 1$), whereas the cross position is assumed as its `off` state ($S_{\mathrm{W},n} = 0$). If the optical signal can reach the upper weight modulator bank of the axons, enclosed between the demultiplexer (DEMUX) and multiplexer (MUX), as is the case in Fig. S1(a) for $S_{\mathrm{X},n} = 1 \wedge S_{\mathrm{W},n} = 1, \forall n$ or in Fig. S1(c) $S_{\mathrm{X},n} = 0 \wedge S_{\mathrm{W},n} = 0, \forall n$, each channel m will be pondered by its designated weight set $W_m = [w_{1,m}, \ldots, w_{N,m}]$, depicted by appropriately colored lines connecting input and output circles in the right-hand side abstractions. Otherwise, if the optical signal reaches a single weight modulator, as is the case in Fig. S1(b) for $S_{\mathrm{X},n} = 1 \wedge S_{\mathrm{W},n} = 0, \forall n$ or in Fig. S1(d) $S_{\mathrm{X},n} = 0 \wedge S_{\mathrm{W},n} = 1, \forall n$, all $m \in [1, M]$ channels will be pondered by identical set of weights $W_0 = [w_{1,0}, \ldots, w_{N,0}]$, represented by grey lines connecting input and output circles in the right-hand side abstractions.

Finally, the output switch $S_{\mathrm{O},n}$ is not controlled independently; its state depends on the path along which the optical signal will arrive to it. Its truth table is given in Table 1 in the main body of the Manuscript and can be summarized through XNOR operation as $S_{\mathrm{O},n} = S_{\mathrm{X},n} \odot S_{\mathrm{W},n}$.

The following modes of operation are supported by PPNN, according to Fig. S1 and Table 1:

a) *multi-neuron* - Each channel λ_m carries corresponding N-element input sequence $x_{n,m}$ where $n \in [1, \ldots, N]$ and $m \in [1, \ldots, M]$ (in total $N \times M$ inputs) which is pondered by a designated set of $N \times M$ weights $w_{n,m}$ and summed across N axons, yielding M outputs y_m. As shown in Fig. S1(a) this translates to M independent neurons with a fan-in of N.

b) *convolutional* - Each channel λ_m carries corresponding N-element input sequence $x_{n,m}$ where $n \in [1, \ldots, N]$ and $m \in [1, \ldots, M]$ (in total $N \times M$ inputs) which is filtered by a single N element set of nominally wavelength-independent weights $w_{n,0}$, yielding M outputs y_m. According to Fig. S1(b) this translates to M parallel neurons with a fan-in of N

whose filter (weight vector) is identical. This mode of operation is commonly used in image recognition tasks or in any other circumstances when inputs are expected to be filtered by an identical kernel.

c) *fully-connected* - All channels λ_m carry identical N-element input sequence $x_{n,0}$ where $n \in [1,\ldots,N]$ (in total N unique inputs) which is pondered by a designated set of $N \times M$ weights $w_{n,m}$, yielding M outputs y_m. As illustrated in Fig. S1(c) this implies that each of N inputs is connected to each of M outputs via a unique connection, concluding to a fully-connected layer. These types of layers are particularly convenient for classification and denoising purposes.

d) *power-saving* - All channels λ_m carry identical N-element input sequence $x_{n,0}$ where $n \in [1,\ldots,N]$ (in total N unique inputs) which is filtered by a single N element set of weights $w_{n,0}$, yielding M identical outputs $y_1 = \ldots = y_M$. From practical point of view, this layer is intended to be used with only one channel being active when sequential operation is required concluding to a power-saving regime ($M-1$ channels are powered off). If all available channels are active, it can be useful for *in-situ* PPNN calibration purposes with respect to wavelength sensitive performance.

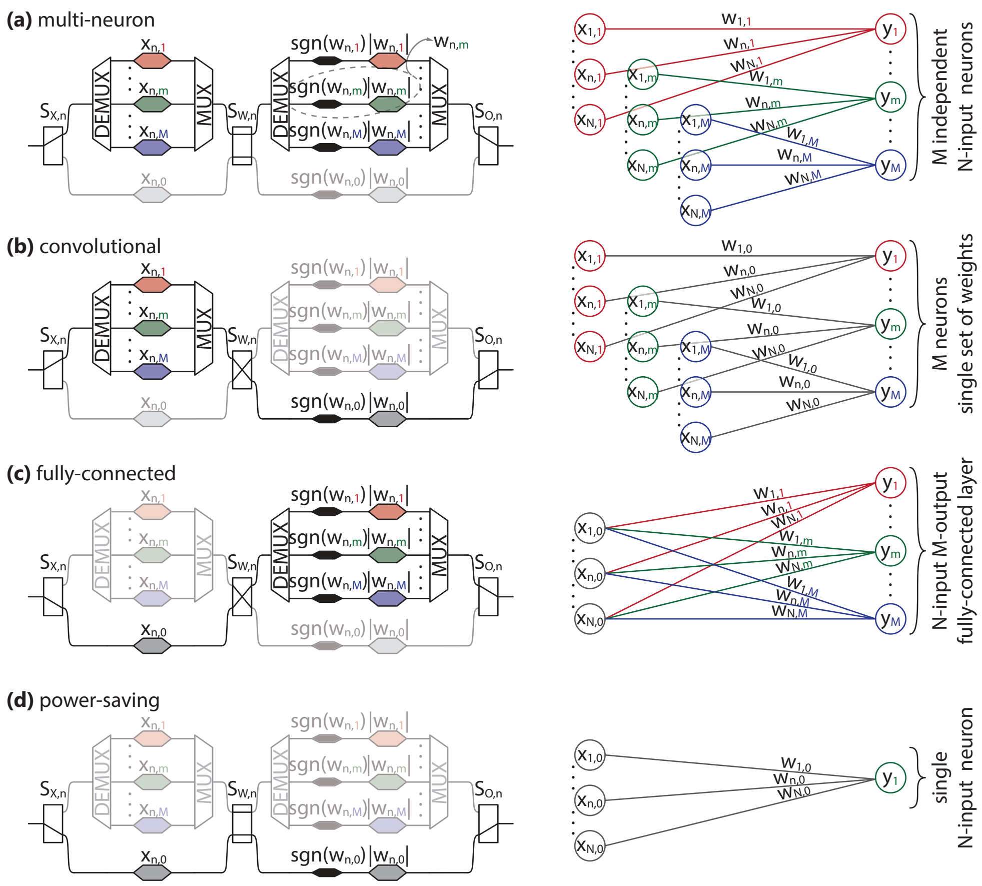

Figure S1. Mapping between the PPNN modes of operation represented through its n-th branch (axon) configuration (left-hand-side) and the corresponding NN layer abstraction (right-hand-side) for: (a) multi-neuron, (b) convolutional, (c) fully-connected (FC), and (d) power-saving arrangement. Semitransparent paths of axons are inaccessible to the optical signal. MUX: multiplexer, DEMUX: demultiplexer

S2 Multichannel PPNN theoretical foundations

The input signal into the PPNN, given by the column-vector E_{LD}, as defined in the main body of the Manuscript, first passes through the 3dB X-coupler, as shown in Fig. 1(a), resulting in the signal entering the bias branch, $E_{B,in}$, and the one entering

the Optical Linear Algebraic Unit (OLAU), $\mathrm{E_{OLAU,in}}$, which read

$$\mathrm{E_{B,in}} = \frac{1}{\sqrt{2}}\mathrm{E_{LD}}\mathrm{e}^{i\pi/2}\,, \tag{S1a}$$

$$\mathrm{E_{OLAU,in}} = \frac{1}{\sqrt{2}}\mathrm{E_{LD}}\,. \tag{S1b}$$

The bias branch acts on an input vector via its diagonal matrix W_b, represented in Fig. 1(c). On the other hand, OLAU requires the signal to pass several stages before the inputs X_n and weights W_n are imprinted. Starting from the input signal defined by equation (S1b), we determine the input into each axon following the 1-to-N splitter depicted in Fig. 1(b). The 1-to-N splitter assumes that the fan-in N is a power of 2 and consists of $\log_2 N$ stages of concatenated 3dB X-couplers where one stage's output is forwarded to the upper ports of the next stage's input, while the lower input ports are kept dark. For each bar-path of the coupler no phase change is introduced to the signal, whereas each cross-path brings a phase shift of $\pi/2$ assuming the coupler is ideal. As the splitting is done equally in terms of power, path taken through the splitting stage will define only the phase accumulated by each signal, resulting in

$$\mathrm{E_{OLAU,in,}}_n = \frac{1}{\sqrt{N}}\left(\mathrm{e}^{i\pi/2}\right)^{k_n}\mathrm{E_{OLAU,in}} = \frac{1}{\sqrt{2}}\frac{1}{\sqrt{N}}\left(\mathrm{e}^{i\pi/2}\right)^{k_n}\mathrm{E_{LD}}\,, \tag{S2}$$

where k_n represents the n-th element of the sequence of Hamming (binary) weights of N[1]

$$\mathrm{K} = [0,1,1,2,1,2,2,3,\ldots,\log_2 N]\,. \tag{S3}$$

Depending on the mode of PPNN operation, each of n axons will either allow for channel-selective imprint of inputs and/or weights, or use a single input and/or weight for all channels. Following the conventions outlined in Table 2 related to input X_n and weight W_n diagonal matrices, prior to entering the N-to-1 combining stage signals will read

$$\mathrm{E_{OLAU,out,}}_n = W_n X_n \mathrm{E_{OLAU,in,}}_n = \frac{1}{\sqrt{2}}\frac{1}{\sqrt{N}}\left(\mathrm{e}^{i\pi/2}\right)^{k_n} W_n X_n \mathrm{E_{LD}}\,. \tag{S4}$$

The outputs from N axons will subsequently interfere within N-to-1 combiner given in Fig. 1(d). The combiner is designed to be a π-rotated copy of the input 1-to-N splitter, where one stage's lower outputs enter the next stages inputs, ensuring in that way identical phase accumulation across all N signals, while the upper outputs of previous stage are discarded. The result of the interference is

$$\mathrm{E_{OLAU,out}} = \frac{1}{\sqrt{N}}\sum_{n=1}^{N}\left(\mathrm{e}^{i\pi/2}\right)^{l_n}\mathrm{E_{OLAU,out,}}_n = \frac{1}{\sqrt{2}}\frac{1}{N}\sum_{n=1}^{N}\left(\mathrm{e}^{i\pi/2}\right)^{l_n+k_n} W_n X_n \mathrm{E_{LD}}\,, \tag{S5}$$

where l_n represents the n-th element of the reversed sequence of Hamming (binary) weights of N

$$\mathrm{L} = [\log_2 N,\ldots,3,2,2,1,2,1,1,0] = \log_2 N - K\,. \tag{S6}$$

Finally, $l_n + k_n$ can be substituted by $\log_2 N$ according to equations (S3) and (S6). The outputs of the bias branch, $\mathrm{E_{B,out}}$, and the OLAU, $\mathrm{E_{OLAU,out}}$, are

$$\mathrm{E_{B,out}} = \frac{1}{\sqrt{2}}W_\mathrm{b}\mathrm{E_{LD}}\mathrm{e}^{i\pi/2}\,, \tag{S7a}$$

$$\mathrm{E_{OLAU,out}} = \frac{1}{\sqrt{2}}\frac{1}{N}\left(\mathrm{e}^{i\pi/2}\right)^{\log_2 N}\sum_{n=1}^{N} W_n X_n \mathrm{E_{LD}}\,. \tag{S7b}$$

The two signals interfere in the last 3dB X-coupler of Fig. 1(a), giving

$$\mathrm{E_{out}} = \frac{1}{2}\left(\mathrm{e}^{i\pi/2}\right)^{1+\log_2 N}\left[W_\mathrm{b}\left(\mathrm{e}^{-i\pi/2}\right)^{\log_2 N} + \frac{1}{N}\sum_{n=1}^{N} W_n X_n\right]\times\mathrm{E_{LD}}\,. \tag{S8}$$

S3 Engineering a non-power-of-2 splitter and combiner

In what follows, we present an algorithm for designing a 1-to-N splitter with an arbitrary number of outputs N based on cascading the stages of X-couplers that are not restricted to equal power splitting and their electric field transfer function is described by the following matrix

$$M = \begin{bmatrix} \sqrt{1-\alpha} & i\sqrt{\alpha} \\ i\sqrt{\alpha} & \sqrt{1-\alpha} \end{bmatrix}, \tag{S9}$$

where α denotes power fraction transmitted to the cross port of the coupler. The flowchart of the algorithm is given in Fig. S2 and it is designed to be resilient to variations in splitting ratio due to fabrication tolerances by limiting α to the range $[1/2, 2/3]$. We verify the algorithm for $N = 9$ and $N = 11$.

The algorithm starts by examining if the splitter can be implemented by cascading the $\log_X N$ identical, smaller-scale unit cells providing 1-to-$N^{1/Y}$ splitting, with $X, Y, \log_X N, N^{1/Y} \in \mathbb{N}$. If so, $X = N^{1/Y}$, otherwise $X = N$ and designing of the 1-to-X splitter is initiated. If X is not a prime number, a further splitting of unit cells to k sub-cells should be done, such that the output port count of a single sub-cell, x_i, is a prime number, $X = x_1^{y_1} x_2^{y_2} \dots x_k^{y_k}$. Following the algorithm, in case of $N = 9$, we have $X = 3$ and $Y = 2$, whereas for $N = 11$ we have $X = 11$ and $Y = 1$, where both X values are prime numbers, yielding $x_1 = 3$ and $y_1 = 1$ in the first case and $x_1 = 11$ and $y_1 = 1$ in the second case. The unit cell (or sub-cell) design requirements are to achieve equal power splitting among all of its outputs, while the induced phase difference will be monitored for each output and compensated by proper engineering of the combining stage.

In designing the sub-cell(s), if the number of outputs, x_i, is even, a 3dB splitter is used with the input forwarded to the upper port and the two outputs, equal in terms of power, recorded at the upper and lower output port, having accumulated phase shifts of 0 and $\pi/2$, respectively. In general, the algorithm would proceed by designing two smaller-scale splitters, each of which would have $x_i = x_i/2$ output ports; however, since the only even prime number is 2, yielding $x_i = 1$, the design of the sub-cell is finished.

Otherwise, if the number of outputs, x_i, is odd, the algorithm starts by bringing the input signal to the upper input of the X-coupler with $\alpha = (x_i + 1)/(2x_i)$, and collecting the signals having a 0-phase change for the upper output and a $\pi/2$ for the lower one. The algorithm proceeds with designing new sub-cells, one with the number of outputs $x_i = (x_i - 1)/2$ and the other with $x_i = (x_i + 1)/2$. It starts by checking if the number of outputs is even or odd and follows the previously outlined procedure which is repeated until $x_i = 1$ is reached.

Applying this method to the arbitrary number of outputs X, where X is a prime number, the number of couplers for 1-to-X cell will be $X - 1$, and the number of stages (cascades) $\lceil \log_2 X \rceil$, implying that the maximum accumulated phase shift per cell will be $\lceil \log_2 X \rceil \pi/2$. For the whole 1-to-$N$ splitter, $(N - 1)$ couplers are needed, arranged in $Y \lceil \log_2 X \rceil$ stages, yielding a maximum phase accumulation of $Y \lceil \log_2 X \rceil \pi/2$.

Applying the developed algorithm to $x_i = 3$, we have the first X-coupler with $\alpha = 2/3$, or the splitting ratio 1/3-to-2/3, followed by another 3dB (or $\alpha = 1/2$) X-coupler connected to the lower port of the initial coupler. The three outputs are equal in terms of power and have accumulated phase shifts of $[0, \pi/2, \pi]$. The full 1-to-N splitter can be realized by concatenating 1-to-X unit cells at each of the outputs of the initial, first-layer's cell. In the case of $N = 9$, we use a total of $N - 1 = 8$ couplers, the powers at the outputs are identical, $1/N = 1/9$ of the input power, whereas the phase accumulation within the n-th axon reads $\exp(ik_n\pi/2)$, where k_n is the n-th element of $k = [0, 1, 2, 1, 2, 3, 2, 3, 4]$.

The combining of the signals leaving the axons is done in an inverse manner, using X-to-1 combiner elementary units, constructed by rotating the splitter elementary unit by π. Signals are forwarded to both inputs of X-coupler, but collected only from the lower output. In this manner, it is ensured that the phase accumulation for the signal coming from the n-th input will read $\exp(il_n\pi/2)$, where l_n is the n-th element of $\lceil \log_2 X \rceil - k$, yielding an overall identical phase accumulation for all signals, ensuring coherence preservation and constructive interference.

When it comes to the unit cell with $x_i = 11$, we split the power as 5/11-to-6/11 by setting $\alpha = 6/11$. We then proceed with the design of two couplers, one with $x_i = 5$ outputs and the other with $x_i = 6$ outputs. The first one splits the input with the power ratio of 2/5-to-3/5, further forwarded to 3dB coupler for the upper and 1-to-3 coupler for the lower port (designed by concatenating 1/3-to-2/3 coupler and another 3dB coupler at the lower output port). The second coupler, used for $x_i = 6$, starts with a 3dB coupler, followed by two 1-to-3 couplers for each of the outputs. The total number of couplers used is $X - 1 = 10$, the total number of stages is $\lceil \log_2 X \rceil = 4$ and the total phase accumulation is 2π. The X-to-1 combiner is designed following the same, previously described approach: rotating the splitter by π and collecting the outputs only from the lower ports.

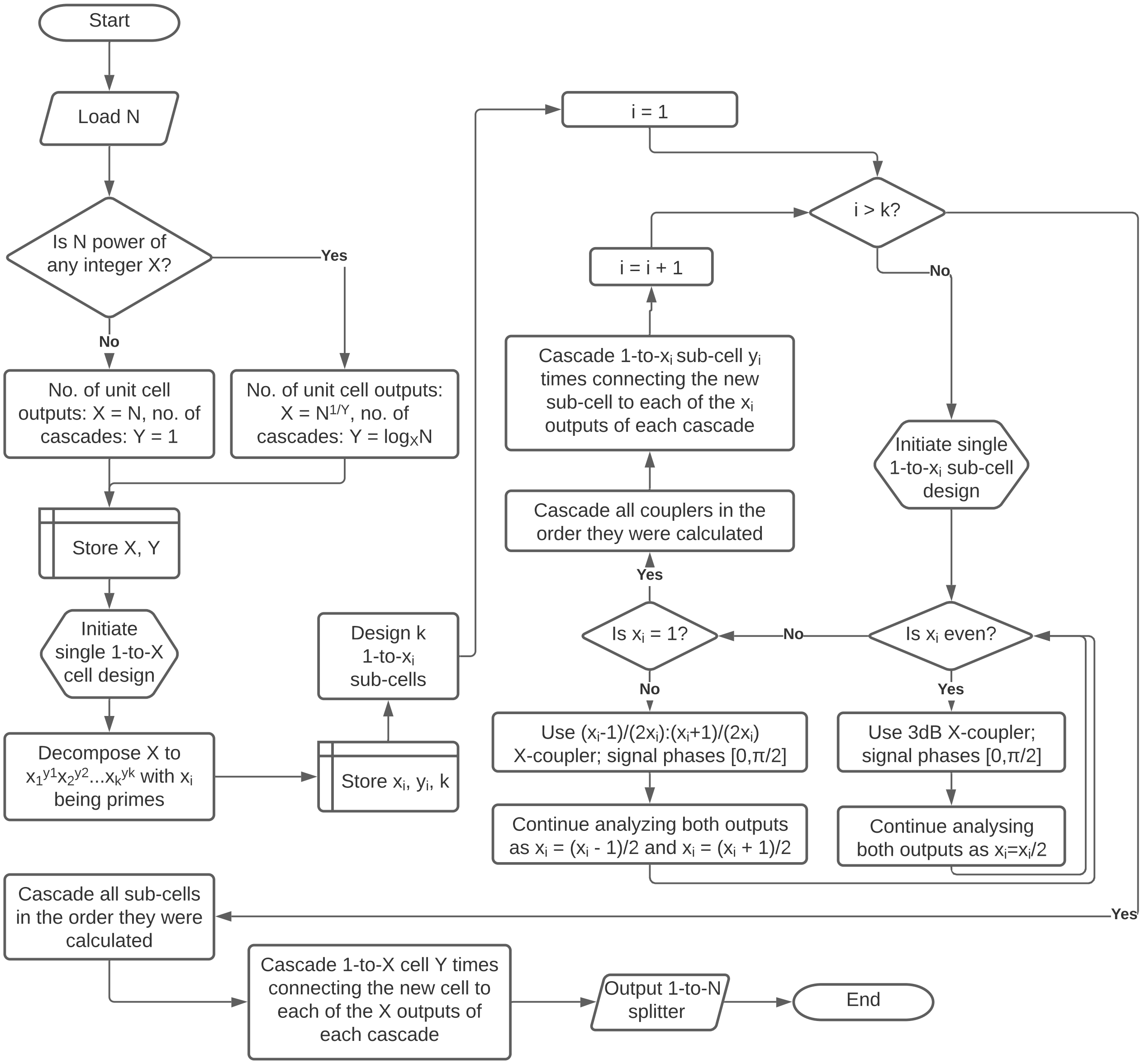

Figure S2. Flowchart of the algorithm for designing power-conserving 1-to-N splitting stage by employing X-couplers. The couplers can have arbitrary splitting ratio defined on the domain $\alpha \in [1/2, 2/3]$ and N does not need to fulfil any particular requirement.

S4 Splitters, combiners and switches

Let us assume that X-coupler has a wavelength dependent transfer function such that (S9) for the m-th channel can be rewritten as

$$M_m = \begin{bmatrix} \sqrt{1-\alpha_m} & i\sqrt{\alpha_m} \\ i\sqrt{\alpha_m} & \sqrt{1-\alpha_m} \end{bmatrix}, \tag{S10}$$

where $\alpha_m = 1/2 + \Delta\alpha_m$ denotes the power splitting ratio of the m-th channel and $\Delta\alpha_m$ its deviation from the targeted value of $1/2$ in case of power-of-2 splitting/combining stages. The deviation can be either positive or negative and is not required to fulfil any particular requirement, except that its magnitude does not exceed $1/2$, i.e., that the X-coupler allows communication between all input and output ports. When operated in splitter mode (having active only one input, E_{in}), the column-vector signals exiting through the two output ports of the X-coupler, $\mathrm{E}_{\mathrm{out,bar}}$ and $\mathrm{E}_{\mathrm{out,cross}}$ will be given as

$$\mathrm{E}_{\mathrm{out,bar}} = \frac{1}{\sqrt{2}}\mathrm{A}_{\mathrm{bar}}\mathrm{E}_{\mathrm{in}}\,, \tag{S11a}$$

$$\mathrm{E}_{\mathrm{out,cross}} = \frac{1}{\sqrt{2}}\mathrm{A}_{\mathrm{cross}}\mathrm{E}_{\mathrm{in}}\mathrm{e}^{i\pi/2}\,, \tag{S11b}$$

where diagonal matrices $\mathrm{A}_{\mathrm{bar}}$ and $\mathrm{A}_{\mathrm{cross}}$ carry the wavelength dependent deviations of splitting ratios

$$\mathrm{A}_{\mathrm{bar}} = \mathrm{diag}\left[\sqrt{1-2\Delta\alpha_1}, \ldots, \sqrt{1-2\Delta\alpha_M}\right], \tag{S12a}$$

$$\mathrm{A}_{\mathrm{cross}} = \mathrm{diag}\left[\sqrt{1+2\Delta\alpha_1}, \ldots, \sqrt{1+2\Delta\alpha_M}\right]. \tag{S12b}$$

On the contrary, when operated in coupler mode (having active both inputs, $\mathrm{E}_{\mathrm{in,bar}}$ and $\mathrm{E}_{\mathrm{in,cross}}$), the column vector of the signal leaving the X-coupler, $\mathrm{E}_{\mathrm{out}}$ reads

$$\mathrm{E}_{\mathrm{out}} = \frac{1}{\sqrt{2}}\left(\mathrm{A}_{\mathrm{bar}}\mathrm{E}_{\mathrm{in,bar}} + \mathrm{A}_{\mathrm{cross}}\mathrm{E}_{\mathrm{in,cross}}\mathrm{e}^{i\pi/2}\right). \tag{S13}$$

Let us also assume that the transfer function of the switch introduces a wavelength dependent loss-penalty originating from non-ideal routing, such that the amount of optical power forwarded to the active port (consult Table 1 in the main body of the Manuscript) is proportional to $s_m \leq 1$, implying that the electrical field of the optical signal passing through the switch gets pondered by $\sqrt{s_m}$. Assuming that the inactive branches of the input or weight banks will have their modulators set to zero-transmission, we can assume that the excess optical power, proportional to $1 - s_m$, will diminish and is not of concern for further analysis. The transfer function of the switch can be given in matrix form

$$\mathrm{S} = \mathrm{diag}\left[\sqrt{s_1}, \ldots, \sqrt{s_M}\right]. \tag{S14}$$

Having three switches in each axon (S_{X}, S_{W} and S_{O}, see Fig. 1(e) in the main body of the Manuscript), and assuming they are identical among themselves and among different axons, the loss-penalty will accumulate to S^3.

Taking (S10)-(S14) into account, we repeat the procedure from Section S2 and find that the signals entering the bias branch and the OLAU read

$$\mathrm{E}_{\mathrm{B,in}} = \frac{1}{\sqrt{2}}\mathrm{A}_{\mathrm{cross}}\mathrm{E}_{\mathrm{LD}}\mathrm{e}^{i\pi/2}\,, \tag{S15a}$$

$$\mathrm{E}_{\mathrm{OLAU,in}} = \frac{1}{\sqrt{2}}\mathrm{A}_{\mathrm{bar}}\mathrm{E}_{\mathrm{LD}}\,. \tag{S15b}$$

After being passed through 1-to-N splitting stage, the signal entering the n-th axon is

$$\mathrm{E}_{\mathrm{OLAU,in},n} = \frac{1}{\sqrt{2}}\frac{1}{\sqrt{N}}\mathrm{A}_{\mathrm{bar}}^{1+l_n}\mathrm{A}_{\mathrm{cross}}^{k_n}\left(\mathrm{e}^{i\pi/2}\right)^{k_n}\mathrm{E}_{\mathrm{LD}}\,, \tag{S16}$$

where k_n and l_n denote the n-th element of the Hamming weight sequence and its reverse, given by (S3) and (S6), respectively. At the output of the n-th axon, accounting for the switch-induced wavelength selective loss, we have

$$\mathrm{E}_{\mathrm{OLAU,out},n} = \frac{1}{\sqrt{2}}\frac{1}{\sqrt{N}}\mathrm{S}^3\mathrm{A}_{\mathrm{bar}}^{1+l_n}\mathrm{A}_{\mathrm{cross}}^{k_n}\left(\mathrm{e}^{i\pi/2}\right)^{k_n}W_nX_n\mathrm{E}_{\mathrm{LD}}\,. \tag{S17}$$

Passing the signals from all axons through N-to-1 combining stage yields

$$\mathrm{E}_{\mathrm{OLAU,out}} = \frac{1}{\sqrt{N}}\sum_{n=1}^{N}\mathrm{A}_{\mathrm{bar}}^{k_n}\mathrm{A}_{\mathrm{cross}}^{l_n}\left(\mathrm{e}^{i\pi/2}\right)^{l_n}\mathrm{E}_{\mathrm{OLAU,out},n} = \frac{1}{\sqrt{2}}\frac{1}{N}\mathrm{S}^3\mathrm{A}_{\mathrm{bar}}^{1+\log_2 N}\mathrm{A}_{\mathrm{cross}}^{\log_2 N}\left(\mathrm{e}^{i\pi/2}\right)^{\log_2 N}\sum_{n=1}^{N}W_nX_n\mathrm{E}_{\mathrm{LD}}\,. \tag{S18}$$

The signal leaving the bias branch reads

$$\mathrm{E}_{\mathrm{B,out}} = \frac{1}{\sqrt{2}}\mathrm{A}_{\mathrm{cross}}W_{\mathrm{b}}\mathrm{E}_{\mathrm{LD}}\mathrm{e}^{i\pi/2}\,. \tag{S19}$$

Finally, the two signals given by (S18) and (S19) interfere in the last X-coupler, giving

$$\mathrm{E}_{\mathrm{out}} = \frac{1}{2}\mathrm{S}^3\left(\mathrm{A}_{\mathrm{bar}}\mathrm{A}_{\mathrm{cross}}\mathrm{e}^{i\pi/2}\right)^{1+\log_2 N}\left(\widetilde{W}_{\mathrm{b}} + \frac{1}{N}\sum_{n=1}^{N}W_nX_n\right)\times\mathrm{E}_{\mathrm{LD}}\,, \tag{S20}$$

where $\widetilde{W}_{\mathrm{b}}$ denotes the bias branch channel-wise transfer matrix accounting for loss balancing and phase alignment, with its m-th element being

$$\widetilde{w}_{\mathrm{b},m} = s_m^{-3/2}\left(\sqrt{1-4\Delta\alpha_m^2}\mathrm{e}^{i\pi/2}\right)^{-\log_2 N} w_{\mathrm{b},m}\,. \tag{S21}$$

S5 Inputs: Amplitude modulator – MZM

In case of input imprinting, we assume that Mach-Zehnder Modulators (MZMs) are voltage controlled, with both of their arms having Phase Shifters (PSs) and that splitting/coupling is ideal in terms of optical power. Induced phase shifts are decomposed to the contribution coming from DC bias voltage, $\phi_{\mathrm{DC},1/2}(V_{\mathrm{DC},1/2},\lambda)$, and modulation RF voltage, $\phi_{1/2}(V_{1/2},\lambda)$, where subscripts 1 and 2 correspond to upper and lower phase shifter, respectively. Assuming push-pull operation, i.e., $V_1 = V_{\mathrm{RF}}$ and $V_2 = -V_{\mathrm{RF}}$, and assuming that the refractive index n dependence on the applied voltage can be represented by an odd function in the 1st order approximation, phase shifts can be written in the following form

$$\phi_{\mathrm{DC},1/2}(V_{\mathrm{DC},1/2},\lambda) = \frac{2\pi}{\lambda}n(V_{\mathrm{DC},1/2},\lambda)L_{\mathrm{DC}}\,, \tag{S22a}$$

$$\phi_1(V_{\mathrm{RF}},\lambda) = \frac{2\pi}{\lambda}n(V_{\mathrm{RF}},\lambda)L = \frac{2\pi}{\lambda}n_0(\lambda)L + \frac{2\pi}{\lambda}\Delta n(V_{\mathrm{RF}},\lambda)L = \phi_0(\lambda)+\Delta\phi(V_{\mathrm{RF}},\lambda)\,, \tag{S22b}$$

$$\phi_2(-V_{\mathrm{RF}},\lambda) = \frac{2\pi}{\lambda}n(-V_{\mathrm{RF}},\lambda)L = \frac{2\pi}{\lambda}n_0(\lambda)L - \frac{2\pi}{\lambda}\Delta n(V_{\mathrm{RF}},\lambda)L = \phi_0(\lambda)-\Delta\phi(V_{\mathrm{RF}},\lambda)\,, \tag{S22c}$$

where L_{DC} and L stand for the lengths of the DC and RF electrodes of PSs, which may be different if separate phase sifters are used, or identical if bias is applied together with the RF signal, whereas $n_0 = n(V=0)$ denotes the refractive index of the material without having the voltage applied and $\Delta n(V) = n(V) - n_0$.

The electric field transfer function of the MZM in push-pull configuration reads

$$t_{\mathrm{MZM}}(V_{\mathrm{DC},1/2},V_{\mathrm{RF}},\lambda) = \cos\left(\frac{2\Delta\phi+\phi_{\mathrm{DC},1}-\phi_{\mathrm{DC},2}}{2}\right)\exp\left(i\frac{2\phi_0+\phi_{\mathrm{DC},1}+\phi_{\mathrm{DC},2}}{2}\right)\,. \tag{S23}$$

Let us assume that the modulator is centered to operate at the wavelength λ_c, which can be either equal to the channel wavelength if a modulator-per-channel is used (as is the case in modes of operation #1 and #2 shown in Fig. S1(a), (b)), or chosen independently if one modulator for several channels is used (as is the case in modes of operation #3 and #4 shown in Fig. S1(c), (d)). In either of the two cases, the transfer function should be optimized to yield the appropriate $x_{n,c}$ value at λ_c and the deviation should be monitored for the remaining wavelengths. Choosing the length of the PS such that $\phi_0(\lambda_c) = 2p_x\pi$, where $p_x \in \mathbb{N}$ and thus eliminating the accumulated phase shift at the central wavelength, we have

$$t_{\mathrm{MZM}}(\lambda_c) = \cos\left[\frac{2\Delta\phi(\lambda_c)+\phi_{\mathrm{DC},1}(\lambda_c)-\phi_{\mathrm{DC},2}(\lambda_c)}{2}\right]\exp\left[i\frac{\phi_{\mathrm{DC},1}(\lambda_c)+\phi_{\mathrm{DC},2}(\lambda_c)}{2}\right]\,. \tag{S24}$$

Assigning the minimum value of the input transfer function to zero RF voltage requires following condition to be met

$$\cos\left[\frac{\phi_{\mathrm{DC},1}(\lambda_c)-\phi_{\mathrm{DC},2}(\lambda_c)}{2}\right]\exp\left[i\frac{\phi_{\mathrm{DC},1}(\lambda_c)+\phi_{\mathrm{DC},2}(\lambda_c)}{2}\right] = 0\,, \tag{S25}$$

which implies

$$\frac{\phi_{\mathrm{DC},1}(\lambda_c) - \phi_{\mathrm{DC},2}(\lambda_c)}{2} = \left(q_1 - \frac{1}{2}\right)\pi\,, \tag{S26}$$

where $q_1 \in \mathbb{Z}$. The simplest approach, by which generality is still not lost, is to choose $q_1 = 0$ and set $\phi_{\mathrm{DC},1}(V_{\mathrm{DC},1}, \lambda_c) + \pi = \phi_{\mathrm{DC},2}(V_{\mathrm{DC},2}, \lambda_c) = \phi_{\mathrm{DC}}(V_{\mathrm{DC}}, \lambda_c)$ transforming equation (S24) to

$$t_{\mathrm{MZM}}(\lambda_c) = \sin\Delta\phi(V_{\mathrm{RF}}, \lambda_c)\exp\left\{i\left[\phi_{\mathrm{DC}}(V_{\mathrm{DC}}, \lambda_c) - \frac{\pi}{2}\right]\right\}. \tag{S27}$$

In order to eliminate the accumulated phase shift at λ_c, we choose the DC voltages such that $\phi_{\mathrm{DC}}(V_{\mathrm{DC}}, \lambda_c) = 2q_x\pi + \pi/2$, where $q_x \in \mathbb{N}$ resulting in

$$t_{\mathrm{MZM}}(\lambda_c) = \sin\Delta\phi(V_{\mathrm{RF}}, \lambda_c)\,, \tag{S28}$$

which will eventually be equal to the input $x_{n,c}$, which we are aiming to imprint at λ_c.

Variations of $\Delta\phi(V_{\mathrm{RF}}, \lambda)$ and $\phi_{\mathrm{DC},1}(\lambda) - \phi_{\mathrm{DC},2}(\lambda)$ with wavelength can be neglected in the following analysis as they are orders of magnitude lower than the variation of either $\phi_0(\lambda)$ or $\phi_{\mathrm{DC},1}(\lambda) + \phi_{\mathrm{DC},2}(\lambda)$, i.e., they are proportional to Δn, as opposed to n. This implies that no significant variation of the transfer function magnitude is anticipated with variation of wavelength; rather, the major contribution will be reflected within the transfer function's phase. This allows us to write, based on equation (S23) and with previously introduced assumptions

$$t_{\mathrm{MZM}}(\lambda) \approx \sin\Delta\phi(V_{\mathrm{RF}}, \lambda_c)\exp\left\{i\left[\phi_0(\lambda) + \phi_{\mathrm{DC}}(V_{\mathrm{DC}}, \lambda) - \frac{\pi}{2}\right]\right\}. \tag{S29}$$

Restricting ourselves to the 1st order approximation, phases ϕ_0 and ϕ_{DC} can be estimated for λ in close proximity of λ_c as

$$\phi_0(\lambda) \approx \phi_0(\lambda_c) + \left.\frac{\partial\phi_0(\lambda)}{\partial\lambda}\right|_{\lambda_c}\Delta\lambda = 2p_x\pi\left[1 - \frac{n_{\mathrm{g}}(\lambda_c)}{n(\lambda_c)}\frac{\Delta\lambda}{\lambda_c}\right], \tag{S30a}$$

$$\phi_{\mathrm{DC}}(\lambda) \approx \phi_{\mathrm{DC}}(\lambda_c) + \left.\frac{\partial\phi_{\mathrm{DC}}(\lambda)}{\partial\lambda}\right|_{\lambda_c}\Delta\lambda = \left(2q_x\pi + \frac{\pi}{2}\right)\left[1 - \frac{n_{\mathrm{g}}(\lambda_c)}{n(\lambda_c)}\frac{\Delta\lambda}{\lambda_c}\right], \tag{S30b}$$

where $\Delta\lambda = \lambda - \lambda_c$ and $n_{\mathrm{g}} = n/(1 + \lambda/n \cdot \partial n/\partial\lambda)$ is the group index of refraction. Introducing equation (S30) to equation (S29) we have

$$t_{\mathrm{MZM}}(\lambda) \approx \sin\Delta\phi(V_{\mathrm{RF}}, \lambda_c)\exp\left[-2i\left(p_x + q_x + \frac{1}{4}\right)\pi\frac{n_{\mathrm{g}}(\lambda_c)}{n(\lambda_c)}\frac{\Delta\lambda}{\lambda_c}\right], \tag{S31}$$

which implies that for a nominal input $x_{n,c}$, only the channel λ_c will have the targeted value imprinted, whereas any other channel m will carry the signal

$$x_{n,m,c} \approx x_{n,c}\exp\left(-i\xi^{(x)}_{m,c}\right), \tag{S32a}$$

$$\xi^{(x)}_{m,c} = 2\left(p_x + q_x + \frac{1}{4}\right)\pi\frac{n_{\mathrm{g}}(\lambda_c)}{n(\lambda_c)}\frac{1}{\lambda_c}(m - c)\Delta\lambda_1\,, \tag{S32b}$$

where the subscript "$\{m,c\}$" denotes that the value $x_{n,m,c}$ is experimental (recorded at channel $m \neq c$) rather than targeted. In equation (S32b), $\Delta\lambda_1 = \lambda_{m+1} - \lambda_m$ denotes the channel spacing (assuming equidistant channels), whereas $p_x = n(V = 0, \lambda_c)L/\lambda_c$ and $q_x = n(V_{\mathrm{DC}}, \lambda_c)L_{\mathrm{DC}}/\lambda_c$ represent the normalized lengths of the RF and DC pads of the phase shifters within the MZM and are restricted to $p_x, q_x \in \mathbb{N}$.

S6 Weights: Amplitude modulator followed by a phase shifter – MZM-PS

Assuming that both arms have thermally-controlled PSs and that splitting/coupling is ideal in terms of power, the MZM's electric field transfer function will depend on different phase shifts in two arms, ϕ_1 and ϕ_2. Adding an additional PS following the MZM, with the phase shift ϕ_3, allows for precise control of the signal's phase, which carries the sign of the weight. The electric field transfer function of the MZM-PS system reads

$$t_{\mathrm{MZM-PS}}(\lambda) = \cos\left(\frac{\phi_1 - \phi_2}{2}\right)\exp\left(i\frac{\phi_1 + \phi_2}{2}\right)\exp(i\phi_3)\,. \tag{S33}$$

MZM operates by being biased at 2θ-point, i.e., having $\phi_1 - \phi_2 = 2\theta$ at the nominal temperature T_0. If θ is chosen to be $\pi/3$, the magnitude of the transfer function at the nominal temperature will be $\cos\theta = 1/2$; otherwise, if $\pi/4$ is chosen, the magnitude will be $\cos\theta = 1/\sqrt{2}$. We impose another condition, $\phi_1 + \phi_2 = 4p_w\pi$ at λ_c, where $p_w \in \mathbb{N}$, eliminating the phase offset at the nominal temperature and giving $\phi_1(T_0,\lambda_c) = 2p_w\pi + \theta$ and $\phi_2(T_0,\lambda_c) = 2p_w\pi - \theta$.

At any point in time, only one phase shifter is being used for adjusting the weight magnitude $|w_{n,c}|$ by increasing its temperature. The lengths of the two PSs within MZM arms are equal and the inherent phase difference is achieved by increasing/reducing the length of the waveguide in the arms by the appropriate amount. Under these assumptions, phases ϕ_1 and ϕ_2 can be written in the following form, if the magnitude of the weight is $|w_{n,c}| \leq \cos\theta$

$$\phi_1(T,\lambda) = \frac{2\pi}{\lambda} n(T_0 + \Delta T, \lambda) L + \theta = \phi(T_0,\lambda) + \theta + \Delta\phi(\Delta T,\lambda)\,, \tag{S34a}$$

$$\phi_2(T_0,\lambda) = \frac{2\pi}{\lambda} n(T_0,\lambda) L - \theta = \phi(T_0,\lambda) - \theta\,, \tag{S34b}$$

or, if $|w_{n,c}| \geq \cos\theta$

$$\phi_1(T_0,\lambda) = \frac{2\pi}{\lambda} n(T_0,\lambda) L + \theta = \phi(T_0,\lambda) + \theta\,, \tag{S35a}$$

$$\phi_2(T,\lambda) = \frac{2\pi}{\lambda} n(T_0 + \Delta T,\lambda) L - \theta = \phi(T_0,\lambda) - \theta + \Delta\phi(\Delta T,\lambda)\,, \tag{S35b}$$

where $\phi(T_0,\lambda) = 2\pi n(T_0,\lambda)L/\lambda$ and $\Delta\phi(\Delta T,\lambda) = 2\pi\Delta n(\Delta T,\lambda)L/\lambda$. Based on equations (S34) and (S35), the sum and the difference of the two phases is

$$\phi_1(\lambda) + \phi_2(\lambda) = 2\phi(T_0,\lambda) + \Delta\phi(\Delta T,\lambda)\,, \tag{S36a}$$

$$\phi_1(\lambda) - \phi_2(\lambda) = 2\theta - \mathrm{sgn}\left(|w_{n,c}| - \cos\theta\right)\Delta\phi(\Delta T,\lambda)\,, \tag{S36b}$$

Substituting equation (S36) to equation (S33) we have

$$t_{\mathrm{MZM-PS}}(\lambda) = \cos\left[\theta - \frac{1}{2}\mathrm{sgn}\left(|w_{n,c}| - \cos\theta\right)\Delta\phi(\Delta T,\lambda)\right] \times \exp\left\{i\left[\phi(T_0,\lambda) + \frac{1}{2}\Delta\phi(\Delta T,\lambda) + \phi_3(\lambda)\right]\right\}. \tag{S37}$$

Incorporating the condition for eliminating the phase offset at the nominal temperature introduced earlier, $\phi(T_0,\lambda_c) = 2p_w\pi$, where $p_w \in \mathbb{N}$, we can equate the transfer function of the MZM-PS system, given by equation (S37), at the central wavelength λ_c with the targeted weight value $w_{n,c}$ and determine the required thermally induced phase shift in MZM arms, as well as in the subsequent standalone PS as follows

$$\Delta\phi(\Delta T,\lambda_c) = 2\,\mathrm{sgn}\left(|w_{n,c}| - \cos\theta\right)\left(\theta - \arccos|w_{n,c}|\right)\,, \tag{S38a}$$

$$\phi_3(\lambda_c) = \frac{1 - \mathrm{sgn}(w_{n,c})}{2}\pi + 2p_s\pi - \frac{1}{2}\Delta\phi(\Delta T,\lambda_c)\,. \tag{S38b}$$

When looking at $\lambda \neq \lambda_c$, variation of $\Delta\phi(\Delta T,\lambda_c)$ with wavelength can be neglected as it is orders of magnitude lower than variation of either $\phi(T_0,\lambda)$ or $\phi_3(\lambda)$, i.e., it is proportional to Δn, as opposed to n, resulting in

$$t_{\mathrm{MZM-PS}}(\lambda) \approx |w_{n,c}| \exp\left\{i\left[\phi(T_0,\lambda) + \frac{1}{2}\Delta\phi(\Delta T,\lambda_c) + \phi_3(\lambda)\right]\right\}. \tag{S39}$$

Restricting ourselves to the 1st order approximation, phases ϕ and ϕ_3 can be estimated for λ in close proximity of λ_c as

$$\phi(\lambda) \approx \phi(\lambda_c) + \left.\frac{\partial\phi(\lambda)}{\partial\lambda}\right|_{\lambda_c} \Delta\lambda = 2p_w\pi\left[1 - \frac{n_g(\lambda_c)}{n(\lambda_c)}\frac{\Delta\lambda}{\lambda_c}\right]\,, \tag{S40a}$$

$$\phi_3(\lambda) \approx \phi_3(\lambda_c) + \left.\frac{\partial\phi_3(\lambda)}{\partial\lambda}\right|_{\lambda_c} \Delta\lambda = \phi_3(\lambda_c)\left[1 - \frac{n_g(\lambda_c)}{n(\lambda_c)}\frac{\Delta\lambda}{\lambda_c}\right]\,, \tag{S40b}$$

where $\Delta\lambda = \lambda - \lambda_c$ and $n_g = n/(1 + \lambda/n \cdot \partial n/\partial\lambda)$ is the group index of refraction. Introducing equation (S40) to equation (S39) and recognizing that in all cases of practical interest $p_s, p_w \gg 1$, we have

$$t_{\mathrm{MZM-PS}}(\lambda) \approx |w_{n,c}| \exp\left[i\frac{1 - \mathrm{sgn}(w_{n,c})}{2}\pi\right] \exp\left[-2i(p_w + p_s)\pi\frac{n_g(\lambda_c)}{n(\lambda_c)}\frac{\Delta\lambda}{\lambda_c}\right]\,, \tag{S41}$$

which implies that for a nominal weight $w_{n,c}$, only the channel λ_c will have the targeted value imprinted, whereas any other channel m will carry the signal

$$w_{n,m,c} \approx w_{n,c} \exp\left(-i\xi^{(w)}_{m,c}\right), \tag{S42a}$$

$$\xi^{(w)}_{m,c} = 2(p_w + p_s)\pi \frac{n_g(\lambda_c)}{n(\lambda_c)} \frac{1}{\lambda_c}(m-c)\Delta\lambda_1, \tag{S42b}$$

where the subscript $\{m,c\}$ denotes that the value $w_{n,m,c}$ is experimental (recorded at $m \neq c$) rather than targeted. In equation (S42b), $\Delta\lambda_1 = \lambda_{m+1} - \lambda_m$ denotes the channel spacing (assuming equidistant channels), whereas $p_w = n(T_0, \lambda_c)L/\lambda_c$ and $p_s = n(T_0, \lambda_c)L_3/\lambda_c$ represent normalized lengths of the PSs within the MZM and the standalone PS, respectively, and are restricted to $p_w, p_s \in \mathbb{N}$.

S7 Signal multiplexing and demultiplexing

As outlined in the main body of the Manuscript, for purposes of (de)multiplexing, Arrayed Waveguide Gratings (AWGs) are used with the assumption of parabolic channel-wise power transfer function. According to the power conservation law, the transfer function of the pass channel reads $T_{\text{AWG}}(0) = (1+2r_{\text{AWG}})^{-1}$ and in the case of suppressed channels we have $T_{\text{AWG}}(\pm\Delta\lambda_1) = r_{\text{AWG}}/(1+2r_{\text{AWG}})$, with r_{AWG} denoting AWG crosstalk in linear terms. The formalism above is valid for both DEMUX and MUX. In case of DEMUX, m-th channel, denoted by subscript, is distributed to the targeted and two adjacent ports, denoted by superscript

$$\frac{1}{\sqrt{1+2r_{\text{AWG}}}}\left\{\sqrt{r_{\text{AWG}}}E^{m-1}_m, E^m_m, \sqrt{r_{\text{AWG}}}E^{m+1}_m\right\}. \tag{S43}$$

These signals get modulated either by $\{x_{n,m-1}, x_{n,m}, x_{n,m+1}\}$ or $\{w_{n,m-1}, w_{n,m}, w_{n,m+1}\}$ depending on the mode of PPNN operation. However, as already shown in Chapters S5 and S6, being detuned from the wavelength for which the modulators are optimized, side channels, indexed by $m \pm 1$, will carry suboptimal input or weight value. In the following analysis we focus on imprinting of inputs in modes of operation #1 and #2, as shown in Fig. S1 (a), (b), recognizing that the same formalism can be applied for weights imprinting in cases #1 and #3, as given in Fig. S1 (a), (b). After demultiplexing, signals are pondered by the corresponding $x_{n,m,c}$ values as follows

$$\frac{1}{\sqrt{1+2r_{\text{AWG}}}}\left\{\sqrt{r_{\text{AWG}}}x_{n,m,m-1}E^{m-1}_m, x_{n,m}E^m_m, \sqrt{r_{\text{AWG}}}x_{n,m,m+1}E^{m+1}_m\right\}. \tag{S44}$$

When reaching the MUX, instead of collecting only the pass channel (at the m-th port), MUX will also collect residuals of the two adjacent ports (indexed $m-1$ and $m+1$ in the superscript):

$$\frac{1}{1+2r_{\text{AWG}}}\left\{r_{\text{AWG}}x_{n,m,m-1}E^{m-1}_m, x_{n,m}E^m_m, r_{\text{AWG}}x_{n,m,m+1}E^{m+1}_m\right\}, \tag{S45}$$

all of which are at the same wavelength λ_m yielding the output electric field

$$\frac{1}{1+2r_{\text{AWG}}}\left[r_{\text{AWG}}(x_{n,m,m-1} + x_{n,m,m+1}) + x_{n,m}\right]E_m. \tag{S46}$$

Two additional sets of approximations can be made: (i) knowing that the crosstalk exists only between adjacent channels, and assuming that the channel spacing $\Delta\lambda_1$ is not large, phase shift due to suboptimal inputs/weights can be neglected implying $x_{n,m,m-1} \approx x_{n,m-1}$ and $x_{n,m,m+1} \approx x_{n,m+1}$, and ($ii$) typical values of $r_{\text{AWG}} \ll 1$ allow to approximate $(1+2r_{\text{AWG}})^{-1} \approx 1 - 2r_{\text{AWG}}$ finally resulting in an experimentally recorded input

$$x^{\text{AWG}}_{n,m} \approx x_{n,m} + r_{\text{AWG}}(x_{n,m-1} - 2x_{n,m} + x_{n,m+1}), \tag{S47}$$

under the constrain $x_{n,0} = x_{n,M+1} = 0$. The same formalism can be applied to weights in modes of operation #1 and #3

$$w^{\text{AWG}}_{n,m} \approx w_{n,m} + r_{\text{AWG}}(w_{n,m-1} - 2w_{n,m} + w_{n,m+1}), \tag{S48}$$

with $w_{n,0} = w_{n,M+1} = 0$, as well as biases in all modes of operation

$$w^{\text{AWG}}_{\text{b},m} \approx w_{\text{b},m} + r_{\text{AWG}}(w_{\text{b},m-1} - 2w_{\text{b},m} + w_{\text{b},m+1}). \tag{S49}$$

with $w_{\text{b},0} = w_{\text{b},M+1} = 0$.

S8 Approximate experimental PPNN matrices

Experimental operation of the PPNN can be described by its transfer function, $\mathrm{Q_e}$, given in diagonal matrix form, similar to the targeted one, $\mathrm{Q_t}$, defined by equation (2) in the main body of the Manuscript

$$\mathrm{Q_e} = \mathrm{diag}[q_{\mathrm{e},1}, \ldots, q_{\mathrm{e},M}] = \widetilde{W}_{\mathrm{e,b}} + \frac{1}{N}\sum_{n=1}^{N} W_{\mathrm{e},n} X_{\mathrm{e},n}\,, \tag{S50a}$$

$$q_{\mathrm{e},m} = \widetilde{w}_{\mathrm{e,b},m} + \frac{1}{N}\sum_{n=1}^{N} w_{\mathrm{e},n,m} x_{\mathrm{e},n,m}\,, \tag{S50b}$$

where quantities indexed by "e" take different form depending on the mode of operation.

S8.1 Multi-neuron

This mode of operation, given in Fig. S1(a), assumes that both in case of inputs and weights designated modulator per channel is used, yielding

$$q_{\mathrm{e},m} = \widetilde{w}_{\mathrm{b},m}^{\mathrm{AWG}} + \frac{1}{N}\sum_{n=1}^{N} w_{n,m}^{\mathrm{AWG}} x_{n,m}^{\mathrm{AWG}}\,. \tag{S51}$$

Substituting equations (S47), (S48) and (S49) to equation (S51) we have

$$\begin{aligned} q_{\mathrm{e},m} \approx q_{\mathrm{t},m} + r_{\mathrm{AWG}} \{ & (\widetilde{w}_{\mathrm{b},m-1} - 2\widetilde{w}_{\mathrm{b},m} + \widetilde{w}_{\mathrm{b},m+1}) \\ & + \frac{1}{N}\sum_{n=1}^{N} \left[(w_{n,m-1} + w_{n,m+1}) x_{n,m} - 4 w_{n,m} x_{n,m} + w_{n,m}(x_{n,m-1} + x_{n,m+1}) \right] \}\,, \end{aligned} \tag{S52}$$

under the constrain $x_{n,0} = x_{n,M+1} = 0$, $w_{n,0} = w_{n,M+1} = 0$ and $\widetilde{w}_{\mathrm{b},0} = \widetilde{w}_{\mathrm{b},M+1} = 0$.

S8.2 Convolutional

Following the Fig. S1(b), in this mode of operation inputs have designated modulator per channel, whereas weights have a single modulator for all channels, resulting in

$$q_{\mathrm{e},m} = \widetilde{w}_{\mathrm{b},m}^{\mathrm{AWG}} + \frac{1}{N}\sum_{n=1}^{N} w_{n,m,c} x_{n,m}^{\mathrm{AWG}}\,. \tag{S53}$$

Substituting equation (S42) to equation (S53) we have

$$q_{\mathrm{e},m} \approx \exp\left(-i\xi_{m,c}^{(w)}\right) \left[\widetilde{w}_{\mathrm{b},m}^{\mathrm{AWG}} \exp\left(i\xi_{m,c}^{(w)}\right) + \frac{1}{N}\sum_{n=1}^{N} w_{n,0} x_{n,m}^{\mathrm{AWG}} \right]. \tag{S54}$$

Following the previously adopted approach while deriving $\mathrm{Q_t}$, where accumulated phase was not taken into consideration for the transfer matrix of the PPNN (see equations (1) and (2) in the main body of the Manuscript), equation (S54) can be rewritten as

$$q_{\mathrm{e},m} \approx \widetilde{w}_{\mathrm{b},m}^{(w),\mathrm{AWG}} + \frac{1}{N}\sum_{n=1}^{N} w_{n,0} x_{n,m}^{\mathrm{AWG}}\,, \tag{S55}$$

where the phase shifters in the bias branch take the responsibility for phase-aligning the signals leaving the OLAU with the signals coming from the bias branch to allow for constructive interference. The diagonal matrix describing the bias branch will now read $\widetilde{W}_{\mathrm{b}}^{(w)} = \widetilde{W}_{\mathrm{b}} \Xi_c^{(w)} = W_{\mathrm{b}} \Xi_c^{(w)} \exp(-i\pi/2)^{\log_2 N}$, where

$$\Xi_c^{(w)} = \mathrm{diag}\left[\exp\left(i\xi_{1,c}^{(w)}\right), \ldots, \exp\left(i\xi_{M,c}^{(w)}\right)\right], \tag{S56}$$

with the m-th element of $\widetilde{W}_{\mathrm{b}}^{(w)}$ being

$$\widetilde{w}_{\mathrm{b},m}^{(w)} = \widetilde{w}_{\mathrm{b},m} \exp\left(i\xi_{m,c}^{(w)}\right) = |w_{\mathrm{b},m}| \exp(i\varphi_{\mathrm{b},m}) \exp(-i\pi/2)^{\log_2 N} \exp\left(i\xi_{m,c}^{(w)}\right). \tag{S57}$$

Substituting equations (S47) and (S49) to equation (S55) we have

$$q_{\mathrm{e},m} \approx q_{\mathrm{t},m} + \widetilde{w}_{\mathrm{b},m}\left[\exp\left(i\xi_{m,c}^{(w)}\right) - 1\right]$$
$$+ r_{\mathrm{AWG}}\left[\left(\widetilde{w}_{\mathrm{b},m-1} - 2\widetilde{w}_{\mathrm{b},m} + \widetilde{w}_{\mathrm{b},m+1}\right)\exp\left(i\xi_{m,c}^{(w)}\right) + \frac{1}{N}\sum_{n=1}^{N} w_{n,0}\left(x_{n,m-1} - 2x_{n,m} + x_{n,m+1}\right)\right], \tag{S58}$$

under the constrain $x_{n,0} = x_{n,M+1} = 0$ and $\widetilde{w}_{\mathrm{b},0} = \widetilde{w}_{\mathrm{b},M+1} = 0$.

S8.3 Fully-connected

This mode of operation, given in Fig. S1(c), exhibits similar behaviour to convolutional mode, having its inputs imprinted by a single modulator for all channels, whereas the weights are controlled on per-channel basis, yielding

$$q_{\mathrm{e},m} = \widetilde{w}_{\mathrm{b},m}^{\mathrm{AWG}} + \frac{1}{N}\sum_{n=1}^{N} w_{n,m}^{\mathrm{AWG}} x_{n,m,c}\,. \tag{S59}$$

Substituting equation (S32) to equation (S59) we have

$$q_{\mathrm{e},m} \approx \exp\left(-i\xi_{m,c}^{(x)}\right)\left[\widetilde{w}_{\mathrm{b},m}^{\mathrm{AWG}}\exp\left(i\xi_{m,c}^{(x)}\right) + \frac{1}{N}\sum_{n=1}^{N} w_{n,m}^{\mathrm{AWG}} x_{n,0}\right]. \tag{S60}$$

Disregarding the accumulated phase, equation (S60) can be rewritten as

$$q_{\mathrm{e},m} \approx \widetilde{w}_{\mathrm{b},m}^{(x),\mathrm{AWG}} + \frac{1}{N}\sum_{n=1}^{N} w_{n,m}^{\mathrm{AWG}} x_{n,0}\,, \tag{S61}$$

where $\widetilde{W}_{\mathrm{b}}^{(x)} = \widetilde{W}_{\mathrm{b}}\Xi_c^{(x)} = W_{\mathrm{b}}\Xi_c^{(x)}\exp(-i\pi/2)^{\log_2 N}$ and

$$\Xi_c^{(x)} = \mathrm{diag}\left[\exp\left(i\xi_{1,c}^{(x)}\right),\ldots,\exp\left(i\xi_{M,c}^{(x)}\right)\right], \tag{S62}$$

and with the m-th element of $\widetilde{W}_{\mathrm{b}}^{(x)}$ being

$$\widetilde{w}_{\mathrm{b},m}^{(x)} = \widetilde{w}_{\mathrm{b},m}\exp\left(i\xi_{m,c}^{(x)}\right) = |w_{\mathrm{b},m}|\exp(i\varphi_{\mathrm{b},m})\exp(-i\pi/2)^{\log_2 N}\exp\left(i\xi_{m,c}^{(x)}\right). \tag{S63}$$

Substituting equations (S48) and (S49) to equation (S61) we have

$$q_{\mathrm{e},m} \approx q_{\mathrm{t},m} + \widetilde{w}_{\mathrm{b},m}\left[\exp\left(i\xi_{m,c}^{(x)}\right) - 1\right]$$
$$+ r_{\mathrm{AWG}}\left[\left(\widetilde{w}_{\mathrm{b},m-1} - 2\widetilde{w}_{\mathrm{b},m} + \widetilde{w}_{\mathrm{b},m+1}\right)\exp\left(i\xi_{m,c}^{(x)}\right) + \frac{1}{N}\sum_{n=1}^{N}\left(w_{n,m-1} - 2w_{n,m} + w_{n,m+1}\right)x_{n,0}\right], \tag{S64}$$

under the constrain $w_{n,0} = w_{n,M+1} = 0$ and $\widetilde{w}_{\mathrm{b},0} = \widetilde{w}_{\mathrm{b},M+1} = 0$.

S8.4 Power-saving

Final, power-saving mode of operation is given in Fig. S1(d). If used with a single channel, no deviation due to either AWG or wavelength-dependent operation of the modulators will exist and the experimental matrix element will be equal to the targeted one. However, if employed in PPNN calibration with all channels active using a single input and a single weight modulator per axon, matrix element will read

$$q_{\mathrm{e},m} = \widetilde{w}_{\mathrm{b},m}^{\mathrm{AWG}} + \frac{1}{N}\sum_{n=1}^{N} w_{n,m,c}x_{n,m,c}\,. \tag{S65}$$

Substituting equations (S32) and (S42) to equation (S65) we have

$$q_{\mathrm{e},m} \approx \exp\left[-i\left(\xi_{m,c}^{(x)} + \xi_{m,c}^{(w)}\right)\right]\left\{\widetilde{w}_{\mathrm{b},m}^{\mathrm{AWG}}\exp\left[i\left(\xi_{m,c}^{(x)} + \xi_{m,c}^{(w)}\right)\right] + \frac{1}{N}\sum_{n=1}^{N} w_{n,0}x_{n,0}\right\}. \tag{S66}$$

Disregarding the accumulated phase, equation (S66) becomes

$$q_{\mathrm{e},m} \approx \widetilde{w}_{\mathrm{b},m}^{(x,w),\mathrm{AWG}} + \frac{1}{N}\sum_{n=1}^{N} w_{n,0}x_{n,0}\,, \tag{S67}$$

where $\widetilde{W}_{\mathrm{b}}^{(x,w)} = \widetilde{W}_{\mathrm{b}}\Xi_c^{(x)}\Xi_c^{(w)} = W_{\mathrm{b}}\Xi_c^{(x)}\Xi_c^{(w)}\exp(-i\pi/2)^{\log_2 N}$ with $\Xi_c^{(x)}$ and $\Xi_c^{(w)}$ being given by equations (S62) and (S56), respectively. The m-th element of $\widetilde{W}_{\mathrm{b}}^{(x,w)}$ reads

$$\widetilde{w}_{\mathrm{b},m}^{(x,w)} = \widetilde{w}_{\mathrm{b},m}\exp\left(i\xi_{m,c}^{(x)}\right)\exp\left(i\xi_{m,c}^{(w)}\right)\,. \tag{S68}$$

Substituting equation (S49) to equation (S67) we have

$$q_{\mathrm{e},m} \approx q_{\mathrm{t},m} + \widetilde{w}_{\mathrm{b},m}\left\{\exp\left[i\left(\xi_{m,c}^{(x)}+\xi_{m,c}^{(w)}\right)\right]-1\right\} + r_{\mathrm{AWG}}(\widetilde{w}_{\mathrm{b},m-1}-2\widetilde{w}_{\mathrm{b},m}+\widetilde{w}_{\mathrm{b},m+1})\exp\left[i\left(\xi_{m,c}^{(x)}+\xi_{m,c}^{(w)}\right)\right]\,, \tag{S69}$$

under the constrain $\widetilde{w}_{\mathrm{b},0} = \widetilde{w}_{\mathrm{b},M+1} = 0$.

As previously noted, if $m = c$, based on equations (S32b) and (S42b), we have $\xi_{c,c}^{(x)} = \xi_{c,c}^{(w)} = 0$. Additionally, setting all bias modulators to the same value, $\widetilde{w}_{\mathrm{b},m} = \widetilde{w}_{\mathrm{b},c}, \forall m$, simplifies equation (S69) to $q_{\mathrm{e},m} \approx q_{\mathrm{t},m}$.

S9 PPNN performance metrics

Let us assume that the number of active channels is given as $M_A \leq M$ and the number of active axons as $N_A \leq N$. Insertion loss (IL) for the bias branch, in units of dB, remains identical for all four modes of operation given in Table 1 in the main body of the Manuscript, whereas the IL of the OLAU depends on the path taken by signal, given by states of the switches $\mathrm{S_W}$ and $\mathrm{S_O}$ as follows

$$\mathrm{IL_B} = 4\mathrm{IL_{MUX}} + 2\mathrm{IL_C} + \mathrm{IL_W} + \mathrm{IL_R^{(B)}}\,, \tag{S70a}$$

$$\mathrm{IL_{OLAU}} = 2(1+\mathrm{S_X}+\mathrm{S_O})\mathrm{IL_{MUX}} + 2(1+\log_X N\lceil\log_2 X\rceil)\mathrm{IL_C} + 3\mathrm{IL_S} + \mathrm{IL_X} + \mathrm{IL_W} + \mathrm{IL_R^{(A)}} + 10\log_{10}(N/N_A)\,, \tag{S70b}$$

where we adopt the following notation for the insertion losses originating from DE/MUX: $\mathrm{IL_{MUX}}$, X-coupler: $\mathrm{IL_C}$, switch: $\mathrm{IL_S}$, input modulators (amplitude only): $\mathrm{IL_X}$, weight modulators (amplitude and phase combined): $\mathrm{IL_W}$, routing waveguides in the bias branch: $\mathrm{IL_R^{(B)}}$ and the axon: $\mathrm{IL_R^{(A)}}$. In (S70b) we assume that the splitting and combining stages are designed according to the algorithm from Fig. S2 with X being the smallest principal integer root of N; if N is a power of 2, $\log_X N\lceil\log_2 X\rceil$ reduces to $\log_2 N$.

The loss of the PPNN as a whole will be dictated by $\mathrm{IL_{OLAU}}$, being the greater of the two given by (S70). Leaving the optical power, or, equivalently, the loss, in the bias branch as is, will allow its proper operation if bias is used only for sign conversion from phase to the amplitude of the electrical field; however, if the bias also carries useful information, the losses in the bias branch and the OLAU should be made equal, which can be achieved either by relying on the T/O MZM used for bias weight amplitude modulation to suppress the excess optical power or by introducing a Variable Optical Attenuator (VOA) in the bias branch with the attenuation equal to

$$\mathrm{IL_{OLAU}} - \mathrm{IL_B} = 2(\mathrm{S_X}+\mathrm{S_O}-1)\mathrm{IL_{MUX}} + 2\log_X N\lceil\log_2 X\rceil\mathrm{IL_C} + 3\mathrm{IL_S} + \mathrm{IL_X} + \mathrm{IL_R^{(A)}} - \mathrm{IL_R^{(B)}} + 10\log_{10}(N/N_A)\,. \tag{S71}$$

Finally, the PPNN loss reads

$$\mathrm{IL_{PPNN}} = 2(1+\mathrm{S_X}+\mathrm{S_O})\mathrm{IL_{MUX}} + 2(1+\log_X N\lceil\log_2 X\rceil)\mathrm{IL_C} + 3\mathrm{IL_S} + \mathrm{IL_X} + \mathrm{IL_W} + \mathrm{IL_R^{(A)}} + 10\log_{10}(N/N_A)\,. \tag{S72}$$

As a comparison, the non-programmable counterpart of PPNN (denoted as dual-IQ), which supports only one channel, has the insertion loss of

$$\mathrm{IL_{dual-IQ}} = 2(1+\log_X N\lceil\log_2 X\rceil)\mathrm{IL_C} + \mathrm{IL_X} + \mathrm{IL_W} + \mathrm{IL_R^{(A)}} + 10\log_{10}(N/N_A)\,, \tag{S73}$$

implying that the penalty introduced by programmability and multi-channel operation reads

$$\Delta\mathrm{IL} = \mathrm{IL_{PPNN}} - \mathrm{IL_{dual-IQ}} = 2(1+\mathrm{S_X}+\mathrm{S_O})\mathrm{IL_{MUX}} + 3\mathrm{IL_S}\,. \tag{S74}$$

Power consumption of the PPNN is dictated by all of its active components, including the Laser Diodes (LDs), assumed to have the optical output power P_{LD} per channel and wall-plug efficiency of η_{wp}, input amplitude modulators ($P_{\mathrm{X}}^{(\mathrm{DC})}$ and $P_{\mathrm{X}}^{(\mathrm{RF})}$),

weight amplitude and phase modulators (P_W) and switches (P_S). Having input modulators biased such that they output 0 at zero RF voltage, (S25), implies that their power consumption will be proportional to the number of active axons N_A and active channels M_A. Similar conclusion can be made for the power consumption of the weights, even though they are biased at 2θ point at the nominal temperature T_0, (S33)-(S35), implying that their transfer function is nonzero if control voltage signal is not applied. Nevertheless, this poses no issue as the signals will already be suppressed by zero transfer function of the input modulators when $N < N_A$, or, will not be launched into the PPNN if $M_A < M$. The total power consumption (in units of mW) can be calculated as

$$P_{\mathrm{PPNN}} = M_A P_{\mathrm{LD}}/\eta_{\mathrm{wp}} + N[1+\mathrm{S_X}(M_A-1)]P_{\mathrm{X}}^{(\mathrm{DC})} + N_A[1+\mathrm{S_X}(M_A-1)]P_{\mathrm{X}}^{(\mathrm{RF})} + N_A[1+\mathrm{S_O}(M_A-1)]P_{\mathrm{W}} + M_A P_{\mathrm{W}} + N(\mathrm{S_X}+\mathrm{S_W}+\mathrm{S_O})P_{\mathrm{S}} + M_A P_{\mathrm{TIA}} + P_{\mathrm{TEC}}\,. \tag{S75}$$

where we also account for the power consumption of the optional Transimpedance Amplifiers (TIAs), P_{TIA}, following the photodiodes (PDs) if immediate detection is mandated by the specific application, as well as the optional temperature controller (TEC), P_{TEC}. Note that these two terms get reduced proportionally to the number of interconnected PPNN layers. The power consumption per active channel is

$$P_{\mathrm{PPNN},m} = \frac{P_{\mathrm{LD}}}{\eta_{\mathrm{wp}}} + N\left[\frac{1}{M_A}(1-\mathrm{S_X})+\mathrm{S_X}\right]P_{\mathrm{X}}^{(\mathrm{DC})} + N_A\left[\frac{1}{M_A}(1-\mathrm{S_X})+\mathrm{S_X}\right]P_{\mathrm{X}}^{(\mathrm{RF})} + N_A\left[\frac{1}{M_A}(1-\mathrm{S_O})+\mathrm{S_O}\right]P_{\mathrm{W}} + P_{\mathrm{W}} + \frac{N}{M_A}(\mathrm{S_X}+\mathrm{S_W}+\mathrm{S_O})P_{\mathrm{S}} + P_{\mathrm{TIA}} + \frac{1}{M_A}P_{\mathrm{TEC}}\,. \tag{S76}$$

As (S75)-(S76) show, there is no power penalty when excess LDs are powered off, $M_A < M$. On the other hand, penalty exists when the number of employed axons is below the maximum one, $N_A < N$, which is attributed to the synchronized switch states in all axons (a penalty that can be alleviated by allowing the switches to be set independently), as well as due to the DC biasing of the input modulators. We note that $P_{\mathrm{X}}^{(\mathrm{DC})}$ can be set to zero if asymmetrical MZMs are used, providing a built-in phase difference of π between the upper and lower MZM branch.

In case of the non-programmable PNN, which supports only one channel, the power consumption amounts to

$$P_{\mathrm{dual-IQ}} = \frac{P_{\mathrm{LD}}}{\eta_{\mathrm{wp}}} + NP_{\mathrm{X}}^{(\mathrm{DC})} + N_A P_{\mathrm{X}}^{(\mathrm{RF})} + (N_A+1)P_{\mathrm{W}} + P_{\mathrm{TIA}} + P_{\mathrm{TEC}}\,. \tag{S77}$$

If PPNN is configured to operate in multi-neuron mode (#1), where $\mathrm{S_X}=\mathrm{S_W}=\mathrm{S_O}=1$, or in power-saving mode (#4), where $M_A=1$, $\mathrm{S_X}=\mathrm{S_O}=0$ and $\mathrm{S_W}=1$, $P_{\mathrm{dual-IQ}}$ and $P_{\mathrm{PPNN},m}$ are comparable and only marginal power-consumption penalty arises in the PPNN case attributed to switches, which is, in mode #1, counterbalanced by the reduction in TEC power consumption on per-channel basis. On the contrary, operating in modes #2 (convolutional) or #3 (fully-connected) allows sharing of the weight or input modulators, driving the power consumption of PPNN below the one of dual-IQ.

It is worth noting that the power consumption of lasers need not be the maximum available; LDs should be biased such that they guarantee enough power at the PPNN output to meet the sensitivity requirements (P_{R}) and the appropriate margin ($\mathrm{IL_M}$) as follows

$$P_{\mathrm{LD}} = P_{\mathrm{R}}10^{(\mathrm{IL_{PPNN}}+\mathrm{IL_M})/10}\,. \tag{S78}$$

Footprint of the PPNN is governed by the number of employed components and the minimum spacing between them. Let us denote the length of one X-coupler with the associated routing waveguides as L_{C}, the length of the whole axon as $L_A^{(\mathrm{PPNN})}$ and the minimum spacing between the waveguides L_Δ. Without accounting for any particular optimization in device placement, we estimate the PPNN area as

$$A_{\mathrm{PPNN}} = \left[2\left(1+\log_X N\lceil\log_2 X\rceil\right)L_{\mathrm{C}} + L_{\mathrm{A}}^{(\mathrm{PPNN})}\right] \times (NM+N+M-1)L_\Delta\,, \tag{S79}$$

where $L_{\mathrm{A}}^{(\mathrm{PPNN})} = 3L_{\mathrm{S}} + 4L_{\mathrm{MUX}} + L_{\mathrm{X}} + L_{\mathrm{W}} + L_{\mathrm{R}}$ accounts the lengths of the switches, L_{S}, 2 pairs of DE/MUXes, L_{MUX}, input, L_{X}, and weight modulators, L_{W}, and the routing waveguides, L_{R}. The footprint per active channel is

$$A_{\mathrm{PPNN},m} = \left[2\left(1+\log_X N\lceil\log_2 X\rceil\right)L_{\mathrm{C}} + L_{\mathrm{A}}^{(\mathrm{PPNN})}\right] \times \left[\frac{M}{M_A}(N+1) + \frac{1}{M_A}(N-1)\right]L_\Delta\,. \tag{S80}$$

In contrast, the area of a non-programmable, single channel neuron reads

$$A_{\mathrm{dual-IQ}} = \left[2\left(1+\log_X N\lceil\log_2 X\rceil\right)L_{\mathrm{C}} + L_{\mathrm{A}}^{(\mathrm{dual-IQ})}\right] \times NL_\Delta\,, \tag{S81}$$

where $L_{\mathrm{A}}^{(\mathrm{dual-IQ})} = L_{\mathrm{X}} + L_{\mathrm{W}} + L_{\mathrm{R}} < L_{\mathrm{A}}^{(\mathrm{PPNN})}$, implying that the added benefit of programmability introduces a penalty along the longitudinal neuron dimension. When it comes the to lateral one, if we assume the best-case scenario for PPNN operation, $M_A = M$, the coefficient pondering L_Δ reduces to $(N+1)+(N-1)/M$, which, in the limiting case of a very large M, yields $N+1$, revealing the always-present penalty in lateral dimension originating from the added benefit of programmability, i.e., the existence of two alternative routes a signal can take within the input and/or weight banks. In a more realistic case, when M is large enough, but not infinite, e.g., of the order of N, the lateral coefficient yields approximately $N+2$. The larger the N, the lesser footprint penalty will exist [$\sim (1+2/N)$]. On the other hand, when operating with a single wavelength (such as in mode #4 where $M_A = 1$), the lateral penalty becomes proportional to M, i.e., the number of channels for which the PPNN was designed.

The throughput of the PPNN in inference applications, measured in Multiply-Accumulate (MAC) operations per second, depends on the bandwidth of the input modulators and the mode in which the network is operated. Assuming the maximum datarate of B_{X}, we find that modes #1 through #3 operate at

$$T_{\mathrm{PPNN}} = M_A N_A B_{\mathrm{X}}\,, \tag{S82}$$

whereas in mode #4 the throughput reduces to $T_{\mathrm{PPNN}}(\#4) = N_A B_{\mathrm{X}}$, which can be deduced also from $M_A = 1$. In other words, the throughput per channel equals $T_{\mathrm{PPNN},m} = N_A B_{\mathrm{X}}$. Finally, the footprint- and energy-efficiency ($\eta_{\mathrm{F}}^{\mathrm{PPNN}}$ and $\eta_{\mathrm{E}}^{\mathrm{PPNN}}$) are defined as the ratios of the throughput and the area and consumed power, respectively, or equivalently, their per-channel values, and can be calculated based on (S82), (S79) and (S75).

References

1. Sloane, N. J. A. The on-line encyclopedia of integer sequences, A000120. published electronically at https://oeis.org (2021).
